# First interspecific multi-parent advanced generation inter-cross (MAGIC) population in *Capsicum* peppers: development, phenotypic evaluation, genomic analysis, and prospects

**DOI:** 10.1093/hr/uhaf182

**Published:** 2025-07-16

**Authors:** Neus Ortega-Albero, Miguel Díaz-Riquelme, Luciana Gaccione, Lorenzo Barchi, Ana Fita, Adrián Rodríguez-Burruezo

**Affiliations:** Institute for the Conservation and Improvement of Valencian Agrodiversity (COMAV), Universitat Politècnica de València, Camino de Vera S/N, 46022 Valencia, Spain; Institute for the Conservation and Improvement of Valencian Agrodiversity (COMAV), Universitat Politècnica de València, Camino de Vera S/N, 46022 Valencia, Spain; Department of Agricultural, Forest and Food Sciences (DISAFA), Plant Genetics, University of Torino, 10095 Grugliasco, Italy; Department of Agricultural, Forest and Food Sciences (DISAFA), Plant Genetics, University of Torino, 10095 Grugliasco, Italy; Institute for the Conservation and Improvement of Valencian Agrodiversity (COMAV), Universitat Politècnica de València, Camino de Vera S/N, 46022 Valencia, Spain; Institute for the Conservation and Improvement of Valencian Agrodiversity (COMAV), Universitat Politècnica de València, Camino de Vera S/N, 46022 Valencia, Spain

## Abstract

This work presents the first eight-way multi-parental advanced generation inter-cross (MAGIC) population in pepper. This interspecific MAGIC population was built with six *Capsicum annuum* accessions and two *C. chinense* accessions, selected for encompassing a representative and wide genetic diversity, and being complementary for morphological, agronomic, and fruit quality traits*.* The population in its third selfing generation has been phenotyped with reliable descriptors and genotyped using genotyping-by-sequencing to assess its overall diversity, homozygosity, parental contributions, and genetic structure. A great variability was found in the phenotyping study, showing many forms of recombination of all the founder lines. Moreover, new phenotypic combinations were found, as well as transgressive inheritance in quantitative traits. The S3 generation contained a balanced distribution of the parental genomes and each S3 individual seemed to contain a unique genomic combination of the founder lines, reaching high homozygosity. In this regard, a preliminary genome-wide association study (GWAS) was performed for highly heritable traits to evaluate the potential of this population for future breeding prospects. Strong associations were found for most traits analysed, like stem pubescence and fruit colour at maturity stage, with associated genes related to response to stress and defence functions; or fruit wall consistency, with associated genes related to lipid metabolism. Our results show that this first *Capsicum* MAGIC population is a valuable genetic resource for research and breeding purposes in peppers, by identifying genomic regions associated with traits of interest and its potential for future GWAS in more complex agronomical and fruit quality traits.

## Introduction

*Capsicum* peppers (*Capsicum* spp.) are among the most profusely grown vegetables worldwide for fresh fruit consumption and as a spice [1] and they encompass a huge variation in morphological and agronomic traits. Among the cultivated species, *C. annuum* L. is the most economically important and diverse. Another cultivated species, *C. chinense* Jacq., displays a volatile profile that enhances fruit flavour and overall quality. *Capsicum chinense* is phylogenetically close to *C. annuum* and sexual crosses are possible among them [2]. In fact, *C. chinense* has been extensively used as donor for *C. annuum* breeding for fruit quality traits [3, 4].

The variation in the *Capsicum* genus offers an outstanding genetic background for breeding commercial *C. annuum*. In this regard, a better understanding of the genetic basis of these traits has paramount importance for predicting and establishing breeding programmes. *Capsicum annuum* has one of the largest genomes of the Solanaceae family, with around 3.5 Gb and between 75 and 80% of repetitive elements (2*n* = 2*x* = 24) [5], considerably larger than other Solanaceae relatives, like tomatoes and eggplants (aubergines) [6, 7]. This is a major reason why elucidating pepper coding regions and genes has been a challenge for decades and molecular tools have been developed later than for other Solanaceae crops [8, 9]. The first draft genomes of *Capsicum* were released in the last decade. Kim *et al*. [8] published the whole-genome sequencing and assembly of *C. annuum* hot pepper ‘Serrano Criollo de Morelos’ (hereafter CM334), reporting a draft genome of 3.45 Gb; and of *C. chinense* accession PI159236, with an estimated genome of 3.2 Gb. In the same year, Qin *et al*. [9] published the genomic sequence of *C. annuum* cv. ‘Zunla-1’ and its wild parent *C. annuum* var. *glabriusculum* ‘Chiltepín’ with assemblies of 3.35 and 3.48 Gb, respectively. However, it was in 2018 when Hulse-kemp *et al*. [5] applied linked-read sequencing to *C. annuum* to generate a highly ordered and more contiguous reference genome of 3.21 Gb.

Usually, genetic mapping has been performed in segregating progenies from bi-parental crosses, with a small number of haplotypes and recombination events. Although these experimental populations allowed to identify many major genes, they are quite limited in terms of recombination events and, hence, the discovery of quantitative trait loci (QTLs) [10]. In this regard, multi-parent advanced generation inter-cross (MAGIC) populations proposed by Mackay and Powell [11] have been developed to gather and explore phenotypic and genetic variability from a group of more than two founder lines to increase mapping resolution and QTL discovery because of the lower linkage disequilibrium (LD) or population structure [12]. MAGIC populations have also been used successfully in early generations to provide a preliminary vision of the population structure and its perspectives, as well as to understand the levels of inter-crossing of founder lines’ genomes and to identify potential genomic regions related to traits [13–15]. They have also been evaluated in later generations to identify genes and mutations responsible for the expression of more complex traits like production and resistance to stresses [16].

MAGIC populations have been developed in some relevant cereals such as maize, wheat, and rice [13, 17, 18], legumes like chickpea and cowpea [19, 20], and some Solanaceae species like tomato and eggplant [14, 15]. However, no MAGIC population has been reported in *Capsicum* peppers to date, and most genome-wide association studies (GWAS) in this genus have been based on collections of accessions or experimental populations limited to two founder parents [21, 22]. In the present work we report the first interspecific MAGIC population in peppers, derived from interspecific crosses of six *C. annuum* genotypes and two *C. chinense* genotypes to integrate a plethora of genetic backgrounds and morphological, agronomic, and fruit quality traits. This population has been phenotyped for qualitative and quantitative traits and genotyped to evaluate the parental marker distribution in the population and to preliminarily explore the occurrence of QTLs related to some highly inheritable traits.

## Results and discussion

### Multi-parent advanced generation population development

**Figure 1:**
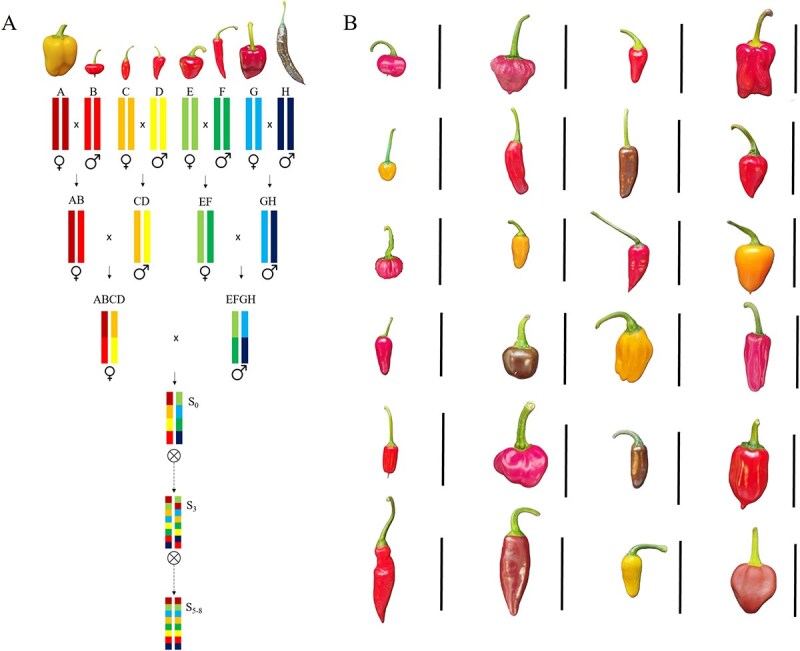
(Left) MAGIC population construction process, founder lines, crosses, and development. A, ‘California Wonder’ (*Capsicum annuum*); B, ‘Ají Dulce’ (*C. chinense*); C, ‘Chile Serrano’ (*C. annuum*); D, ‘Ecu-994’ (*C. chinense*); E, ‘Bola’ (*C. annuum*), F, ‘Serrano Criollo de Morelos’ (*C. annuum*); G, ‘Piquillo’ (*C. annuum*); H, ‘Pasilla Bajío’ (*C. annuum*). (Right) Illustrative representation of the variation among the S3 progeny.

Six genotypes of *C. annuum* and two genotypes of *C. chinense* were selected for their interesting phenotypic traits as founder lines for developing the first interspecific MAGIC pepper population. Following the scheme of Fig. 1, a total of 420 eight-way hybrid individuals, of which 284 derived from ABCD as a mother and 136 derived from EFGH as a mother. The S3 MAGIC population comprised 350 individuals obtained following a single-seed descent (SSD) programme. The introduction of multiple founders from two different species with high genetic and phenotypic diversity enabled the accumulation of a plethora of recombinant events, increasing mapping accuracy of interesting genes [12]. The loss of 70 progenies occurred during the first and second selfing generations, which might be due to the inclusion of two *C. chinense* founder genotypes, despite being used as male parents (B and D). In this regard, previous MAGIC populations have been reported to lose progenies during the first developmental generations as a result of genome rearrangements causing infertility or non-viable hybrids, as occurred with eggplant MAGIC population which included a wild related species [14]. In fact, after three selfing generations, the current S3 population has reached high percentages of pollen fertility, as will be explained in the next section.

### Phenotypic variation and inter-trait correlations

#### Qualitative traits

Most evaluated traits showed variation among the founder lines, like nodal anthocyanin, stem pubescence, leaf shape, flower position, fruit colour, fruit shape parameters, and pedicel persistence. A new phenotype was described for fruit shape in ‘Ají Dulce’ (phenotype ‘Disc’). Differences between *C. annuum* and *C. chinense* included absence of nodal anthocyanins, two flowers per axil, and purple filament colours, only found in *C. chinense* parents. Very few traits were found to be monomorphic (or almost) in the parent lines, including stem colour, neck at the base of the fruit, fruit cross-section corrugation, and anthocyanin spot in the intermediate stage of the fruit (Supplementary Data Table S1).

F_1_ hybrids showed phenotypes intermediate between those of the corresponding parents, the phenotype of one of the parents and even some new phenotypes (Supplementary Data Table S1). The findings relating to stigma exsertion, fruit colour in mature fruits, and fruit shape indicated the dominance of triangular shapes, red colour at the fully ripe stage, due to described recessive alleles for yellow and brown colours, and the exsertion of the stigma, as a wild trait which favours cross-pollination in nature against inserted stigmas [23, 24]. A few cases of new phenotypes were detected in some F_1_ crossings. For example, intermediate leaf density and dark green leave in F_1_ ‘Piquillo’ × ‘Pasilla’, which came from sparse-density and green-leafed parents, or the lanceolate leaves and erect flowers of F_1_ ‘California’ × ‘Ají Dulce’, whose parents had deltoid and ovoid-shaped leaves and pendant and intermediate flowers (Supplementary Data Table S1). The occurrence of new phenotypes has been pointed out by evolutionary biologists during interspecific hybridization, due to genome recombination of varieties and species together with genetic and epigenetic changes [25].

As expected, the S3 progeny showed huge variation for all the studied traits, and the occurrence of new phenotypes was recorded in some traits. Some qualitative traits showed balanced segregation of the different phenotypes, in proportion to their occurrence in the founder lines, like nodal anthocyanin, stem pubescence, leaf density, leaf colour, leaf shape, number of flowers per axil, or flower position (Supplementary Data Table S1). In other cases, the segregation of the different phenotypes was different from that observed in the founder parents, like anther colour or stigma exsertion, with 30% of each phenotype in the S3 population, while one phenotype appeared just in one parent line (Supplementary Data Table S1), confirming the dominance phenomena observed in the F_1_ hybrids for e.g. stigma exsertion. Finally, some cases of new phenotypes, not present in the founder lines, were recorded in traits like stem colour, filament colour, anthocyaninic spots in the intermediate-stage fruit, fruit colour at the mature stage, or fruit shape at pedicel attachment, which suggests the occurrence of genetic complementation or specific recombinations that produced these phenotypes hidden under recessive and complementation effects [18, 20, 26]. Qualitative traits were also evaluated in the S4 generation, confirming, in most traits, the distribution observed in the earlier S3 generation (Supplementary Data Table S1). This can be observed in traits like stem pubescence, leaf colour, and fruit colour at intermediate stage. Only very few more complex traits, like fruit shape or fruit section corrugation, showed slight differences in the phenotype distribution among individuals, which could be due to the different number of evaluated lines in S4 compared with S3. In fact, the expression observed for qualitative traits in individual lines of the S4 generation coincided with that previously observed in the corresponding S3 lines, suggesting that these traits, at least phenotypically, were already fixed in S3.

#### Quantitative traits

All the quantitative traits showed great variation among the founder lines, confirming that they encompass great phenotypic diversity. The most variable traits among founder lines seemed to be stem length, fruit length, width and weight, and seed weight (Supplementary Data Table S2). *Capsicum chinense* showed considerable differences compared with *C. annuum*, like lower fruit weight, pedicel length, fruit shape triangle, and seed weight values. A few traits, like proximal and distal fruit blockiness, fruit shape triangle, and number of locules, showed little variation (Supplementary Data Table S2). Generally, morphological pollen viability showed the highest values compared with biological viability values, germinative viability being the one with the lowest values. These results agree with other results previously reported in *Capsicum* [2]. Founder lines’ viabilities were found to be considerably high, with the only exception of ‘California Wonder’ (Supplementary Data Table S2).

As observed for qualitative traits, F_1_ hybrids showed different performance depending on the trait. In some cases, an intermediate value between the corresponding founder lines was observed, e.g. leaf length, leaf width, and fruit length (Supplementary Data Table S2). For other traits, the phenotype in the F_1_ was closer to one of the corresponding parents, e.g. fruit width, pedicel length, or number of fruit locules (Supplementary Data Table S2). Other traits showed transgressive values, like stem length, internode length, fruit weight, and pollen viability (Supplementary Data Table S2). Pollen viability was particularly low in interspecific F_1_ hybrids between *C. annuum* and *C. chinense*, which is probably due to deleterious phenomena during genome rearrangements [27]. These events are key to understanding the difficulties that interspecific experimental populations face during the first generations, even losing some non-viable lineages.

Generally, the distribution of the traits in the S3 population was within the ranges of variation of the founder lines, as found for internodal length, leaf length and width, fruit length and width, and number of locules (Supplementary Data Table S2). Nevertheless, a great diversity among the S3 population was found, resulting in the appearance of transgressive individuals for most traits. This was observed in internode length, with a coefficient of variation (CV) of 58%, internode number (CV = 30%), leaf length (CV = 17%), leaf width (CV = 19%), pedicel length (CV = 36%), locule number (CV = 18%), and especially in fruit weight, with CV = 102% (Supplementary Data Table S2). These recombination effects have also been reported by other authors in multi-parent populations (MPPs) of several species, including studies about transgressive events [13, 20, 26]. In this regard, new trait combinations and heterosis in fruit traits like fruit weight have been previously observed in interspecific *C. chinense* × *C. annuum* descendant populations [28, 29]. Pollen morphological viability showed relatively high values in the S3 progeny, with a mean value of 41% and a maximum value of 97%. Viability was lower in biochemical and germinative measurements, as expected, with means of 33 and 11%, respectively, although very high values were observed in some individuals, with 96 and 69%, respectively.

**Figure 2:**
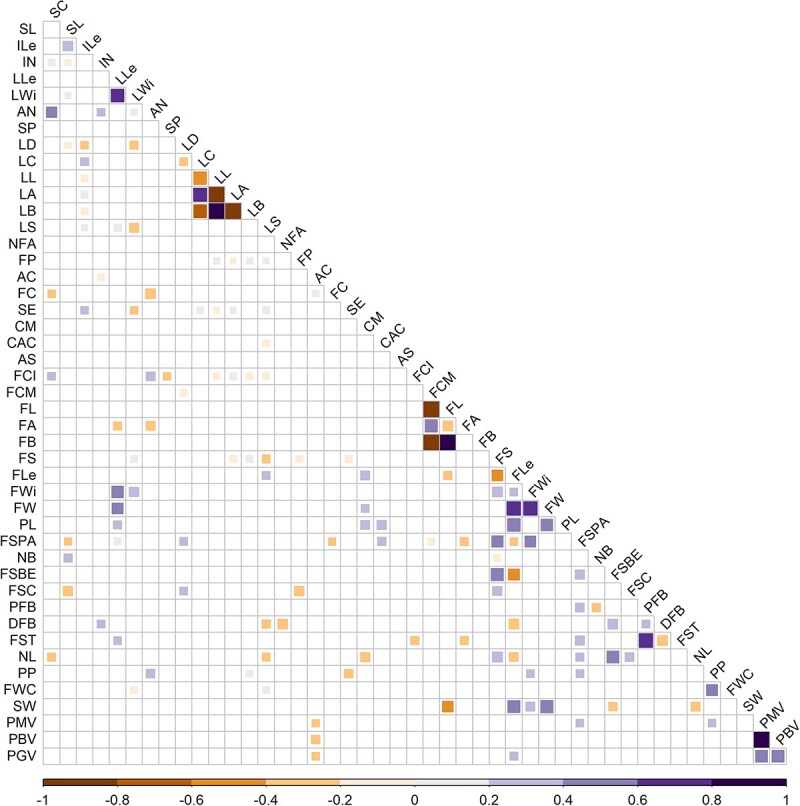
Statistically significant inter-trait correlations with correlation coefficients (Pearson *P* < 0.05), with a size scale for the magnitude of the correlation and a colour scale for significance.

According to our findings, the S3 MAGIC progeny look very useful to study the recombination of the traits. Values of the S4 population have been studied in most traits, confirming the distribution observed in the S3 generation (Supplementary Data Table S2). Most quantitative traits maintained the mean values displayed by the S3 individuals. Thus, the evaluation of the S4 progeny was a check point to confirm that the phenotypic distribution was maintained for most traits among both generations. However, the overall variation observed in some quantitative traits made us decide to evaluate only qualitative traits in the preliminary GWAS analysis.

#### Inter-trait correlations

Significant inter-trait correlations (Pearson *P* < 0.05) were detected considering all the studied populations (i.e. S3 generation, founder lines, and F_1_ and F_1_ × F_1_ hybrids) (Fig. 2). Strong correlations were found within the same group of descriptors. CIE L*a*b* colour coordinates were highly correlated with qualitative colour descriptors for both leaf and fruit, indicating that the values obtained in the CIE L*a*b* space are accurate indicators of the visual classification. High correlations were found between plant descriptors, like the positive correlation between stem colour and nodal anthocyanin, or between plant height and internode length. Fruit traits showed the highest number of correlations among them. For instance, fruit length and fruit width showed high positive correlations with fruit weight, and peduncular persistence seemed to be related to consistent fruit walls, meaning that the softer the fruit, the lower the persistence, indicating common processes associated with fruit ripening and senescence (Fig. 2). Also, biochemical and morphological pollen viabilities as well as germinative viability showed strong positive correlation values. Finally, correlations were also found between different groups of descriptors. As an example, positive correlations were observed between leaf length and fruit weight and between leaf length and fruit length, indicating that plants with larger leaves tend to have bigger fruits, as well as the contrary, i.e. plants with smaller leaves usually produce small or light fruits. This has previously reported in *Capsicum* wild types like ‘Chiltepín’ (*C. annuum* var. *glabriusculum*) and cultivated ‘Cayenne’ types (*C. annuum* var. *annuum*) [30].

#### Principal component analysis

**Figure 3:**
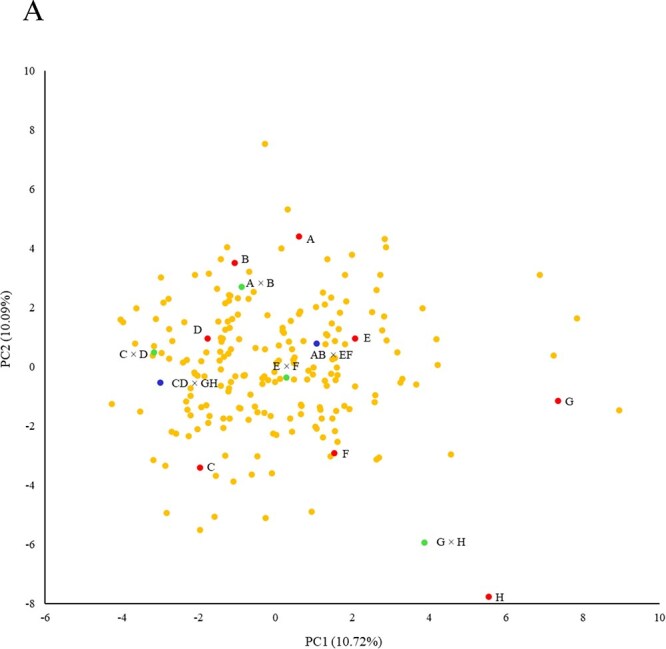
First (PC1) and second (PC2) principal components, representing founder lines (red points), F_1_ (green) and F_1_ × F_1_ hybrids (blue), and the S3 progeny (orange), analysed with phenotypic data.

The first and second principal components explained 10.5 and 9.1% of the observed variation, respectively (Fig. 3). Founder lines appeared mainly located in the borders of the cloud of S3 individuals, indicating that the offspring integrated and displayed most phenotypic variation of the parents. F_1_ hybrids were distributed close to their corresponding founder lines, while F_1_ × F_1_ hybrids were located in the centre of the cloud of the S3 population and were separated by PC1. Finally, some S3 lines appeared apart from the main cloud, even surpassing the founder lines, with extreme phenotypic values in some traits (Fig. 3).

### Genotyping-by-sequencing results

#### Sequencing and SNP calling

Sequencing the S3 MAGIC lines produced 900 million raw paired-end reads and ~110 Gb of data. After de-multiplexing, cleaning and trimming, clean reads were aligned to the reference pepper genome CM334 (Serrano Criollo de Morelos version 1.55) [8]. A total of 1 197 532 polymorphic sites were identified after SNP calling and basic filtering distributed over the 12 chromosomes, and 37 748 were selected for further analysis.

Then, the 1 197 532 polymorphic sites obtained after SNP calling and basic filtering were used for calculating population homozygosity. Homozygosity values for the selected founder lines ranged from 99.1% in ‘Serrano Criollo de Morelos’ to 99.68% in ‘Chile Serrano’, ‘Bola’, and ‘Pasilla Bajío’. All the founder lines of the population showed >99% homozygosity, as they have been selected and bred through generations of auto-pollination. Regarding the F_1_ hybrids, the homozygosity values dropped to 96.87% for the interspecific *C. annuum* × *C. chinense* hybrids, the ones showing the lowest values, indicating that interspecific crosses might result in a higher degree of genomic recombination. This high homozygosity in the F_1_ hybrids suggests that even though phenotypically the founder lines differ remarkably, there is a huge amount of shared genetic background.

Homozygosity values in the S3 progeny ranged from 97.7 to 99.8% and showed a mean value of 99.38%, indicating that after three selfing generations there was fast evolution of fixation in the population. Moreover, although including the *C. chinense* genetic background might have caused some genome deconstruction and reordering and the generation of miss-crossing during meiosis, our S3 population does not apparently show further difficulties in recovering homozygous individuals [27].

#### Genetic relationships and structure of the S3 generation

##### Principal component analysis

**Figure 4:**
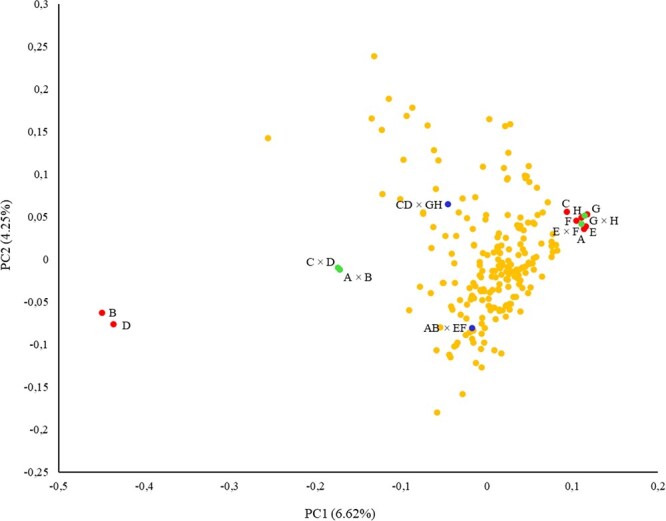
First (PC1) and second (PC2) principal components, representing founder lines (red points), F_1_ (green) and F_1_ × F_1_ hybrids (blue), and the S3 progeny (orange), analysed with GBS data.

After quality filtering (missing data <15% and mean minimal read depth >7, missing data per site <25%, minor allele frequency (MAF >5%) 37 748 SNPs were selected to assess the genetic relationship between the S3 lines and the founder lines. It should be noted that the two first components of the principal component analysis (PCA) only explained 10.87% of the variation (Fig. 4), indicating the huge genetic variability encompassed by our S3 MAGIC population. *Capsicum annuum* parent genotypes appeared clustered in the positive area of the PC1, while *C. chinense* genotypes appeared in the opposite location of PC1, confirming genetic differences between both genetic strains. In this sense, commercial peppers ‘California Wonder’ and ‘Bola’ were located together and slightly separated from the other genotypes, especially ‘Serrano’ peppers, which are considered the closest cultivated types to wild *C. annuum* var. *glabrisculum* i.e. the least domesticated *C. annuum* var. *annuum* [31].

In the case of F_1_ hybrids, *C. annuum* intra-specific hybrids clustered with *C. annuum* genotypes due to the shared genomic background, while interspecific *C. annuum* × *C. chinense* hybrids appeared in the middle of both parent taxa, indicating that these hybrids embedded, in a balanced way, part of their parent genomes (Fig. 4). Also, the F_1_ × F_1_ hybrids were located in the PC1 halfway between inter- and intra-specific F_1_ hybrids, indicating, again, a balanced admixture of the founder genomes in the offspring, in agreement with our findings in the phenotypic characterization. Nevertheless, both selected F_1_ × F_1_ separated considerably along PC2, indicating also considerable differences at the genomic level between these hybrids. Finally, the S3 progeny appeared widely distributed in the PCA, although in terms of PC1 the cloud of individuals was located closer to the *C. annuum* group of parents, as expected in an offspring that should encompass 75% of these genomes versus 25% of the *C. chinense* background. Furthermore, the S3 progeny distributed widely in PC2, much wider than observed in the founder lines, F_1_ and F_1_ × F_1_ hybrids (Fig. 4). Such an outbreak of diversity, particularly in PC2, indicates new recombination events in the MAGIC population.

##### Parental contributions

**Figure 5:**
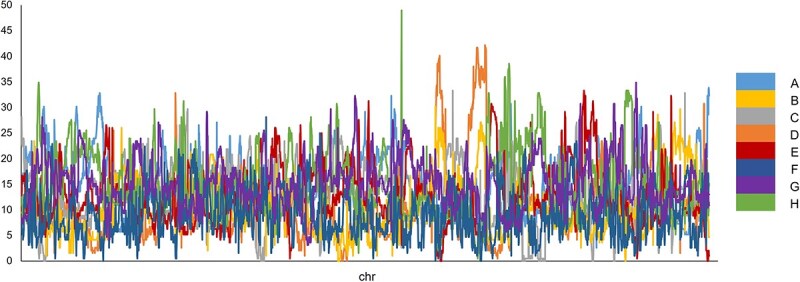
Genome position (*x*-axis) and the percentage of founder contribution (*y*-axis) across the S3 population. The scale on the right shows the colours associated with each founder.

The parental genomic contributions were calculated for the S3 population with the good-quality SNPs. Our results showed that all the parental genomes were represented in, at least, a 10% of the S3 lines, indicating a well-balanced reorganization of all the founder lines (Fig. 5). The representation of the *C. annuum* species was, on average, 78.5%, distributed as 14.2% of ‘California Wonder’, 12.4% of ‘Chile Serrano’, 12.7% of ‘Bola’, 10.1% of ‘Serrano Criollo de Morelos’, 14.7% of ‘Piquillo’, and 14.5% of ‘Pasilla Bajío’. The *C. chinense* genome was present in 21.5%, distributed as 10.8% ‘Ají Dulce’ and 10.6% ‘Ecu-994’. This distribution agrees with the expected values of 12.5% of each founder line, and *C. chinense* genomes were represented in almost 25%. These results agree with the results in Fig. 4: the cloud of the S3 lines displays genetically in a region that is placed in the half-side closer to *C. annuum*, but not overlapping*,* suggesting a mixture of 25% *C. chinense* and 75% *C. annuum*.

##### Dendrogram and kinship matrix

No clear relationships or clusters were observed in the phylogenetic tree among the S3 lines as they appeared widely distributed, in agreement with the findings of the PCA (Supplementary Data Fig. S1A). On the contrary, lines were equally distributed, and the founder lines were placed between the descendants, including *C. annuum* and *C. chinense*, although both species were genetically distant according to the SNP data (Supplementary Data Fig. S1A). Moreover, S3 lines showed more similarities among them and the founder lines than F_1_ and F_1_ × F_1_ hybrids, which showed differentiated genomes compared with the S3 population. Furthermore, the kinship matrix showed that S3 lines represented unique recombination events, as although genetic similarities were found between groups of individuals, no specific clusters were observed (Supplementary Data Fig. S1B). These results confirmed the results of the PCA, indicating that each individual has a unique genetic pattern resulting from the recombination of the founders’ genomes. Therefore, the whole S3 population appears to encompass wide and well-balanced genetic diversity with a lack of genetic structure, as reported in other MAGIC populations of Solanaceae species, like tomato and eggplant [14, 16].

##### Admixture analysis

**Figure 6:**
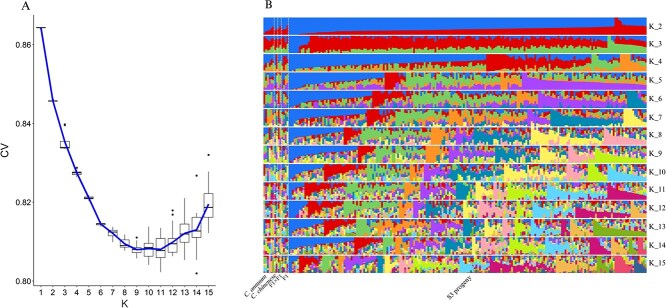
Admixture structure analysis for the MAGIC pepper population including founder lines, F_1_ hybrids, F_1_ × F_1_ hybrids, and 200 S3 individuals. (A) Cross-validation plot to estimate the best-fitting *K* value. (B) Structure of the population.

An admixture analysis was performed to elucidate the genetic patterns and structure of the S3 population. The estimated cross-validation error indicated that *K* = 9–11 were the best statistical models to fit the available SNP data (Fig. 6A). Considering the *K =* 9 model as the simplest one, each individual of the S3 population is made of an admixture of different genomic regions, confirming the results obtained in the kinship matrix (Fig. 6B). Very few individuals showed no admixture or recombination or even similar genome patterns. Also, nine genetic subpopulations fitted quite accurately the eight founder lines, confirming a balanced genome and allelic admixture. High diversity has been found for admixture and substructure in MAGIC populations. For instance, other MAGIC populations, like chickpea, have reached up to 13 subpopulations in the admixture analysis [20], indicating that this analysis is highly dependent on the underlying genome complexity, crossed species, etc., while MAGIC populations of sorghum have shown four subpopulations or groups [26].

### Genotype-wide association study

#### Linkage disequilibrium

A diagram of *r^2^* against physical distances showed a pattern of LD decay that allowed us to establish the inter-marker separation in 500 kb pairs, when the *r^2^* magnitude was reduced to *r^2^* > 0.2 (Supplementary Data Fig. S2). LD generally has great variation depending on the chromosome, as reported by Thudi *et al*. [20], and the point at which LD decays to *r^2^* < 0.2 is also very variable when two markers are considered independent.

Reported LDs were also variable depending on the species; for instance, chickpea showed a mean LD of 1.2 Mb, while barley had an LD decay value around 19 Mbp, and there was an extremely low 40 kb for a 19-way sorghum MAGIC population [20, 26]. LD decay tends to decrease in each generation; the next generations of this MAGIC population will be used to narrow the genetic basis of QTL associated with traits, thus establishing candidate genes.

#### QTL mapping and analysis

**Figure 7:**
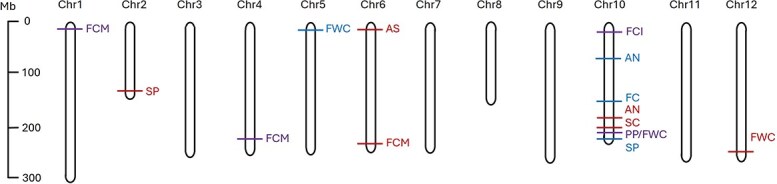
Distribution of the QTLs in a pepper physical map. Coloured bars show the location of the QTLs found for each trait and each statistical model (red, BLINK; blue, MLMM; purple, both models). SC, stem colour; AN, nodal anthocyanin; SP, stem pubescence; FC, filament colour; AS, anthocyanin spot; FCI, fruit colour at intermediate stage; FCM, fruit colour at mature stage; PP, pedicel persistence; FWC, fruit wall consistency.

Significant QTLs were found for nine of the qualitative traits analysed, using BLINK (Bayesian-information and linkage-disequilibrium iteratively nested keyway) and MLMM (multiple loci mixed model) statistical models (Supplementary Data Table S3; Supplementary Data Fig. S3). Based on our data, a higher number of QTLs were found with the BLINK model, although some QTLs were also reported using the two statistical models. This result has also been reported in another GWAS analysis [26], with a higher number of QTLs obtained with BLINK than MLMM. As expected for genetically simple traits, all the QTLs related to the corresponding trait were located on the same chromosome, with the only exception of (i) stem pubescence, where QTLs were located on chromosomes 2 and 10, (ii) anthocyanin spot in intermediate stage fruit, with QTLs on chromosomes 4 and 6, (iii) fruit colour at maturity stage on chromosomes 1, 4, and 6, and (iv) fruit wall consistency with QTLs on chromosomes 5, 10, and 12 (Fig. 7).

Once the significant QTLs for plant-related traits were identified, the associated genes in these regions were investigated (Fig. 7). For stem colour several genes were identified on chromosome 10; however, the most remarkable finding corresponded to the esterase/lipase gene *CPRD49*, presumably related to lipid metabolism (Supplementary Data Table S3). In this regard, tissue pigmentation has previously been associated with globular lipid inclusions and proteins associated with these lipid molecules, embedded with anthocyanins in chromoplasts [32, 33]. In the case of nodal anthocyanin, several genes related to the response and adaptation to changes in temperature have been found on chromosome 10, like *BTB/POZ* domain-containing protein, *HSP17*, and *APRR7,* which are involved in protein modification (Supplementary Data Table S3). In this regard, in agreement with our findings, anthocyanin accumulation has recently been associated with protein modification and gene regulation [34]. We also identified a thylakoid membrane protein gene (*TERC*) associated with nodal anthocyanin, and anthocyanin transport into the vacuoles through activation of transport systems has been reported previously [35]. Our results for colouration patterns in plant organs agree with previous reports in *Capsicum*, indicating the existence of a major gene on chromosome 10 determining pigmentation in the plant, foliage, and flowers [36]. Candidate genes on chromosome 10 need an in-depth study for their function in anthocyanin accumulation in plant tissues. For stem pubescence, genes related to defence were detected on chromosomes 2 and 10, e.g. *R1B-23* and zinc finger CCCH domain-containing protein 14 (Supplementary Data Table S3). These results confirm previous reports indicating QTLs related to trichome accumulation in the stem located on chromosomes 2 and 10 in peppers [37]. Publications about this trait are scarce or absent, although some authors proposed that hair density could be a determinant of plant protection against different biotic and abiotic stresses like cold and drought Moles *et al*. [38]. Moreover, regions on chromosome 2 of *Capsicum* have been related to resistance to pathogens as well as tolerance to abiotic stresses [39, 40].

Regarding flower traits, only filament colour showed significant QTLs on chromosome 10 (Supplementary Data Table S3). These QTLs included genes related to phenol and photosynthetic metabolism and the production of isoflavone formonotenine, e.g. *D7OMT* isoflavone 7-O-methyltransferase and *HEMA1* glutamyl-tRNA reductase 1. These findings suggest that accumulation of phenolic compounds as precursors could be a determinant of the final pigmentation of the filament with purple anthocyanins and reinforce the idea that chromosome 10 accumulates many genes related to pigmentation of the plant and also flower tissues [36, 37].

Finally, significant QTLs were found for fruit descriptors like anthocyanin spot, fruit colour at the intermediate stage, fruit colour at the fully ripe stage, pedicel persistence, and fruit wall consistency (Fig. 7). All QTLs reported for anthocyanin spot in immature fruits were located on chromosome 6 and most genes were associated with defence against pathogens, especially, genes for late blight resistance (*R1B-14, R1A-4* or *R1A-10*) (Supplementary Data Table S3). For fruit colour at the intermediate stage, several QTLs related to cell metabolism, like development or protein modification, were found on chromosome 10, e.g. *SD25* G-type lectin S-receptor-like serine/threonine-protein kinase or *TBL39* protein trichome birefringence-like 39 (Supplementary Data Table S3). In this regard, anthocyanin accumulation has been reported to increase cold tolerance [41], and gene family *CaMYB*, transcription factors regulating development and stress responses, has been reported to accumulate anthocyanins and increase cold tolerance in purple peppers and to be located on chromosome 10 [42–44]. Also, gene *Ca3GT* has been described on chromosome 10 for determining fruit colour at the unripe stage, indicating that fine mapping of the chromosomal regions reported in our work would lead to the same gene [45]. On the whole, our study detected a strong association of QTLs related to response to biotic and abiotic stress (including response to temperature changes) with tissue colour traits, like nodal anthocyanin and unripe fruit colour, indicating that presumably simple traits might be modulated by environmental conditions. Furthermore, we have confirmed the existence of a hot-spot of genes on chromosome 10 determining anthocyanin accumulation in plant organs, flowers, and fruits; and we have proposed new candidate genes for future validation.

A large number of significant QTLs were found for fruit colour at the fully ripe stage, including regions on chromosomes 1, 4, and 6 (Fig. 7). Although in the first studies on inheritance and gene control, fruit colour was described as qualitative or controlled by a few major genes, several later studies have confirmed that variation in colour and intensity can be determined in a quantitative manner, involving many regulating genes [46]. Some of the identified genes in this work were related to fruit development, ripening, and pigment accumulation, like ethylene-responsive proteinase inhibitor 1, alpha-trehalose-phosphate synthase 7, and capsanthin/capsorubin synthase, the last one previously reported in peppers by Jeong *et al*. [23] (Supplementary Data Table S3). Our results regarding chromosomes 1 and 4 coincide with phytoene synthase *CaPSY1* and *CaPSY3* genes described by Wei *et al*. [47] in the same genomic regions for fruit colour in ripe fruits in *Capsicum*. Other QTLs found for fruit colour were highly related to plant development, like cyclin-dependent kinase C-1, protein Fantastic Four 3 or ankyrin repeat-containing protein *BDA1*. Finally, QTLs related to lipid metabolism and hormone response and signalling were also associated with fruit colour at the mature stage, like phosphopantothenoylcysteine decarboxylase and lipid transfer protein GPI-anchored 2 associated with lipid metabolism, and protein *RALF*-like 5, protein ethylene insensitive 3 and vesicle-associated membrane protein 714, associated with hormone signalling.

For pedicel persistence and fruit wall consistency, polygalacturonases were the most remarkable associated genes, in agreement with the findings of Zhai *et al*. [48] (Supplementary Data Table S3). These genes have been preliminarily located in regions of chromosome 10 of *Capsicum* [49, 50]*.* Fruit wall consistency also showed several genes related to lipid metabolism distributed along all the genome. Moreover, some interesting functions were found related to glucose metabolism and responses to biotic and abiotic stresses for fruit wall consistency (Supplementary Data Table S3). Overall, these results indicate that chromosome 10 is a hot spot of genes determining fruit ripening, a key trait for post-harvesting quality improvement.

### Conclusions

In this work we present the first MAGIC interspecific population achieved in *Capsicum* as a valuable tool for future breeding and genetic studies in peppers. A wide phenotypic diversity has been observed within the population, increasing the available *Capsicum* resources. Also, unique genetic patterns have been found in the population, indicating a plethora of recombinant processes, but representing at the same time a well-balanced distribution of the eight parental genomes in the developing MAGIC population. Moreover, with this MAGIC population, genomic regions and candidate genes have been preliminarily associated with the highly inheritable traits of stem colour, nodal anthocyanin, stem pubescence, filament colour, anthocyanin spots in the fruit, fruit colour at the intermediate and mature stages, pedicel persistence, and fruit wall consistency to show the potential of this population. These findings will be exhaustively investigated, and candidate genes will be confirmed for these traits in further experiments. Moreover, future generations of the MAGIC population will be used to also define candidate genes for more complex traits in pepper breeding, like plant architecture, root traits, volatile profile, and accumulation of bioactive compounds in fruit as well as responses to biotic and abiotic stresses.

## Materials and methods

### Development of the MAGIC population

Founder lines of the MAGIC population included six *Capsicum annuum* L. genotypes, corresponding to four cultivated genotypes (‘Bola’, ‘California Wonder Yellow’, ‘Pasilla Bajío’, and ‘Piquillo’) and two semi-wild types (‘Chile Serrano’ and ‘Serrano Criollo de Morelos’); and two *C. chinense* Jacq. genotypes (‘Ají Dulce’ and ‘Ecu-994’) (Fig. 1). These lines were chosen to maximize phenotypic, agronomic, and genetic diversity in the progenies. Among other traits, *C. annuum* lines like ‘California Wonder’ and ‘Piquillo’ were selected because of their high commercial value, with thick fruit flesh. Also, ‘Piquillo’ has an improved root architecture, showing good enzymatic activity in the soil and great absorption, which are good indicators of adaptation to organic and low-input conditions [51]. ‘Pasilla Bajío’ is one of the pepper varieties with the highest accumulation in fruit of antioxidants like ascorbic acid (vitamin C), carotenoids, and flavonoids [52, 53]. ‘Bola’, on the contrary, has been selected for its precocity in fructification, thin flesh, and high water content, and improved production traits. ‘Chile Serrano’ and ‘Serrano Criollo de Morelos’ (cv SCM334), have been selected for their plant architecture, showing a higher plant with open branches, enabling easier harvest, as well as for being the wildest representation of the *C. annuum* taxon, and well known for resilience to hard pedoclimatic conditions [54, 55]. *Capsicum chinense* ‘Ají Dulce’ and ‘Ecu-994’ were added as founder lines of this MAGIC population because they have shown a different volatile profile in fruits compared with *C. annuum* varieties, improving fruit quality and flavour, including pungency as a high added value. Moreover, these accessions had shown a satisfactory crossability in previous work compared with other cultivated peppers and they have been proposed as gene donors for *C. annuum* breeding for many traits of interest, like capsaicinoid content, resistances to multiple pests, and introgressing multiple-flower traits to improve yield [2, 3, 31, 56–58]. These varieties have been used as male parents in their corresponding F_1_ crossings to prevent cytoplasmic deleterious phenomena (dwarfism or virus-like syndrome) that may appear when *C. chinense* is used as the female [2]. F_1_ crosses were made with individual plants of each founder line, previously selected for their highest homozygosis after several selfing processes and genotyping-by-sequencing (GBS) analysis [31]. Only ‘Serrano Criollo de Morelos’ has been selected for resistance purposes, carrying the *Me1* genes responsible for resistance to *Meloidogyne arenaria* and other genetic factors for resistance to *Phytophthora capsici.*

Since 2016, four parental crosses were performed between the accessions as indicated in Fig. 1. Then, F_1_ hybrid ‘California Wonder’ (A) × ‘Ají Dulce’ (B) and F_1_ ‘Chile Serrano’ (C) × ‘Ecu-994’ (D) were crossed to obtain 175 four-way individuals (ABCD). On the other branch, F_1_ hybrid ‘Bola’ (E) × ‘Serrano Criollo de Morelos’ (F) and F_1_ ‘Piquillo’ (G) × ‘Pasilla Bajío’ (H) were crossed to obtain 160 four-way hybrids (EFGH). Then, the two double hybrids were intercrossed in reciprocal crosses to obtain the eight-way or quadruple hybrid generation (Fig. 1). As a result, 420 unique lines were obtained to constitute the S0 generation. These lines were then propagated following an SSD procedure for three generations to obtain an S3 generation of 350 lines in the 2021/22 season.

**Table 1:** List of the traits considered, corresponding abbreviation, IPGRI descriptor number, and unit/scale (if applicable)

**Descriptor**	**Abbreviation**	**IPGRI number**	**Unit/scale**
**Plant descriptors**			
Stem colour	SC	7.1.2.2	1 = green, 2 = green with purple stripes, 3 = purple
Stem length	SL	7.1.2.9	mm
Internode length	ILe		mm
Internode number	IN		
Mature leaf length	LLe	7.1.2.18	mm
Mature leaf width	LWi	7.1.2.19	mm
Nodal anthocyanin	AN	7.1.2.3	1 = green, 3 = light purple, 5 = purple, 7 = dark purple
Stem pubescence	SP	7.1.2.5	1 = sparse, 3 = intermediate, 5 = dense
Leaf density	LD	7.1.2.13	3 = sparse, 5 = intermediate, 5 = dense
Leaf colour	LC	7.1.2.14	1 = yellow, 2 = light green, 3 = green, 4 = dark green 5 = light purple, 6 = purple, 7 = variegated
Leaf colour lightness	LL		0 = black to 100 = white
Leaf colour green/red value	LA		Negative = green, to positive = red
Leaf colour blue/yellow value	LB		Negative = blue, to positive = yellow
Leaf shape	LS	7.1.2.15	1 = deltoid, 2 = ovate, 3 = lanceolate
			
**Inflorescence/flower descriptors**			
Number of flowers per axil	NFA	7.2.1.2	
Flower position	FP	7.2.1.3	3 = pendant, 5 = intermediate, 7 = erect
Anther colour	AC	7.2.1.8	1 = white, 2 = yellow, 3 = pale blue, 4 = blue, 5 = purple
Filament colour	FC	7.2.1.10	1 = white, 2 = yellow, 3 = green, 4 = blue, 5 = light purple, 6 = purple
Stigma exsertion	SE	7.2.1.12	3 = inserted, 5 = same level, 7 = exserted
Calyx margin	CM	7.2.1.15	1 = entire, 2 = intermediate, 3 = dentate
Calyx annular constriction	CAC	7.2.1.16	0 = absent, 1 = present
			
**Fruit descriptors**			
Anthocyanin spots at intermediate stage	AS	7.2.2.2	0 = absent, 1 = present
Fruit colour at intermediate stage	FCI		3 = light green, 5 = green, 7 = dark green
Fruit colour at mature stage	FCM	7.2.2.6	1 = white, 2 = lemon-yellow, 3 = pale orange-yellow, 4 = orange-yellow, 5 = pale orange, 6 = orange, 7 = light red, 8 = red, 9 = dark red, 10 = purple, 11 = brown, 12 = black
Mature fruit colour lightness	FL		0 = black to 100 = white
Mature fruit colour green/red value	FA		Negative = green, to positive = red
Mature fruit colour blue/yellow value	FB		Negative = blue, to positive = yellow
Fruit shape	FS	7.2.2.7	1 = elongate, 2 = almost round, 3 = triangular, 4 = campanulate, 5 = blocky
Fruit length	FLe	7.2.2.8	mm
Fruit width	FWi	7.2.2.9	mm
Fruit weight	FW	7.2.2.10	g
Pedicel length	PL	7.2.2.11	mm
Fruit shape at pedicel attachment	FSPA	7.2.2.13	1 = acute, 2 = obtuse, 3 = truncate, 4 = cordate, 5 = lobate
Neck at base of fruit	NB	7.2.2.14	0 = absent, 1 = present
Fruit shape at blossom-end	FSBE	7.2.2.15	1 = pointed, 2 = blunt, 3 = sunken, 4 = sunken and pointed
Fruit cross-sectional corrugation	FSC	7.2.2.17	3 = slightly corrugated, 5 = intermediate, 7 = corrugated
Proximal fruit blockiness	PFB		
Distal fruit blockiness	DFB		
Fruit shape triangular	FST		
Number of locules	NL	7.2.2.18	
Pedicel with ripe fruit persistence	PP	7.2.2.20.1	3 = slight, 5 = intermediate, 7 = persistent
Fruit wall consistency	FWC		0 = absent, 1 = present
**Pollen/seed descriptors**			
Pollen morphological viability	PMV		%
Pollen biochemical viability	PBV		%
Pollen germinative viability	PGV		%
Weight of 100 seeds	SW	7.3.5	g

Seeds of S3 lines, founder genotypes, the original F_1_ hybrids, and two representative F_1_ × F_1_ hybrids as controls were sown in seedling trays with commercial substrate after disinfection with 30% bleach for 30 min and irrigated with tap water. Trays were maintained at room temperature and watered every 3 days. Once plants reached the four-leaf stage, they were transplanted into 30-cm diameter pots, grown under glasshouse-controlled conditions, trained with vertical strings, drip-irrigated and fertigated following agronomic recommendations for peppers.

### Phenotyping trait evaluation and statistics

Phenotypic variation in unique S3 individuals of the MAGIC population was determined in spring and summer 2023 under greenhouse experimental conditions in the Universitat Politècnica de València (València, Spain) with 47 morphological descriptors of *Capsicum*, corresponding to plant [56], inflorescence/flower [46], fruit [5], pollen [13], and seed [16] traits (Table 1). Also, a collection of 200 S4 individuals were phenotyped in spring and summer 2024 under the same conditions with a selection of descriptors for plant [46], inflorescence/flower [46], fruit [34], pollen [13], and seed [16] traits to check the reproducibility and consistency of the data among both generations. Most descriptors were obtained from Bioversity International [59]. Also, other common traits used in pepper breeding characterization and/or registration, like internode number, internode mean length, fruit wall consistency, and pollen morphological, biological and germinative viability, were added. Fruit shape was also estimated through three ratios: proximal fruit blockiness, distal fruit blockiness, and fruit shape triangle. Proximal fruit blockiness was calculated as the ratio between highest fruit part width (mm) and the centre of the fruit (mm). Distal fruit blockiness was calculated as the ratio between the lowest fruit part width (mm) and the centre of the fruit (mm). Finally, fruit shape triangle was measured as the ratio between highest fruit width (mm) and lowest fruit part width (mm). Fruit wall consistency was estimated by applying pressure to the fruit pericarp with the fingers. Flexible pericarps that retained the original shape were considered consistent, while fruits with permanent deformation or bruising were considered non-consistent.

Pollen morphological and biological activity was measured in a light microscope, with pollen samples from four flowers per plant dyed with 2,3,5-triphenyl tetrazolium chloride (TTC) and kept in darkness for 15 min [60]. Turgid pollen grains in the case of morphological analysis and dyed turgid grains in the case of biological analysis were counted in four fields per sample, with a total magnification of ×100 and referred to total grains. Finally, for pollen germination viability, pollen samples were stirred for 5 hours in a solution of H_3_BO_3_ 100 ppm and 10% sucrose, dyed with acetocarmine with 1% glycerine jelly and incubated as described in Díez *et al*. [61]. Four fields per sample were also observed, with a total magnification of ×100 and viability was estimated as the proportion of pollen grains germinated referred to total grains.

Three measurements for each S3 individual were made for qualitative traits, including plant, flower, and fruit traits. Regarding quantitative traits, three measurements were taken per plant for plant, flower, and seed descriptors; five measurements were made for leaf descriptors; and ten measurements were made for fruit descriptors. Leaf and fruit colour were measured using a Minolta CR-300 colorimeter (Minolta Corporation, Osaka, Japan) and expressed according to CIE L*a*b* 1976 space. Finally, 100-seed weight was measured with an electronic balance (PLJ 600-2GM, Kern & Sohn GmbH, Balingen, Germany).

Statistical analyses were performed using R software [62]. Outliers were removed from phenotypic data and correlations between traits were calculated using the Pearson coefficient with statistical packages [63]. Moreover, mean, standard deviations and variation coefficient were estimated for each trait in the studied genotypes with Statgraphics Centurion XVII software (StatPoint Technologies, Warrenton, VA, USA).

### DNA extraction, sequencing, and mapping, and SNP calling

Leaf tissue samples were obtained from individual plants of a random selection of 200 S3 lines among the previously phenotyped lines, representative of the S3 MAGIC population, together with the founder lines, and F_1_ and F_1_ × F_1_ hybrid individual plants at the four-leaf stage. DNA was extracted using the SILEX method [64]. GBS lsibrary construction and sequencing were performed by The Elshire Group Ltd [65], with the following changes: 100 ng of genomic DNA was used, 3.6 mg of total adapters, digestion with ApeKI enzyme, and amplification with 18 PCR cycles. DNA libraries were sequenced with the Illumina Novaseq 6000 platform using paired-end 150 bp reads. Kevin-Murray’s AX-demux was used for de-multiplexing raw reads and removing barcodes [66]. BWA-MEM with default parameters but avoiding multiple-mapping reads [67] was used to align de-multiplexed reads to the reference *C. annuum* genome CM334 (version 1.55) [8]. BAM files were used for SNP calling and to select minimum mapping quality SNPs (*Q* > 30) with GATK haplotype caller 4.1.9 [68]. Afterwards, good-quality SNPs (missing data <15% and mean minimal read depth >7) were retained with VCFtools [69] for further analyses. Homozygosity was calculated for all the genotypes with VCFtools [69]. The distribution of the parental markers in the population and the parental contribution was calculated with R package HaploBlocker [70].

### Genetic diversity and structure analyses

For elucidating phylogenetic relationships within the MAGIC population, low-quality SNPs (missing data per site >25% and MAF <5%) were removed with bcftools [71] and PLINK2 [72]. To have an illustrative description of the genetic variability of the S3 population and to assess the possible occurrence of a cluster effect and the founder lines, a PCA was first performed with R package SNPrelate [73]. Further, a phylogenetic tree and a kinship matrix were created with IQ-TREE2 [74] and SNPrelate [73], consecutively, to visualize genetic relationships and the genetic distribution of the S3 population. An admixture analysis was run with AdminPipe v2.0 [75] to elucidate the level of genomic mixing of the parents in the MAGIC population, with the following parameters: number of subpopulations (*K*) ranging from 1 to 10, and each *K* run with 16 replicates submitted to pong software [76] to elucidate the best statistical run.

### Genome-wide association study

Phenotypic and genotypic data were used to perform a GWAS with GAPIT (version 3) [77]. In view of the number of individuals that constitute the S3 MAGIC population, only traits expected to be coded by a few genes and previously described as highly inheritable were chosen for the GWAS analysis. Genetic control and regulation of supposedly more complex traits will be further studied in the next S5–S6 generations, when the population is expected to be fully fixed throughout the genome. For the association study, MLMM and BLINK were conducted and corrected with the Bonferroni and false discovery rate (FDR) methods [78] at 0.05 significance level. PopLDDecay [79] was used to determine LD and haplotype structure. The LD correlation coefficient (*r*^2^) was used to establish the intervals between significant independent SNPs with a limit value of 0.2 and distance of 5000 kbp. Candidate genes were retrieved from the CM334 (version 1.55) reference genome [8] with BEDtools [80].

## Supplementary Material

Web_Material_uhaf182

## Data Availability

The genomic raw data underlying this article are available in the Sequence Read Archive (SRA) of the National Institutes of Health (NIH).
