# Supplementary material for: First interspecific multi-parent advanced generation inter-cross (MAGIC) population in Capsicum peppers: development, phenotypic evaluation, genomic analysis, and prospects
Source: Hortic Res. 2025 Jul 16;12(10):uhaf182. doi: 10.1093/hr/uhaf182 (PMC12537016; doi:10.1093/hr/uhaf182)
Supplement: Web_Material_uhaf182 [file web_material_uhaf182.zip › Supplementary Figure 1.pdf]

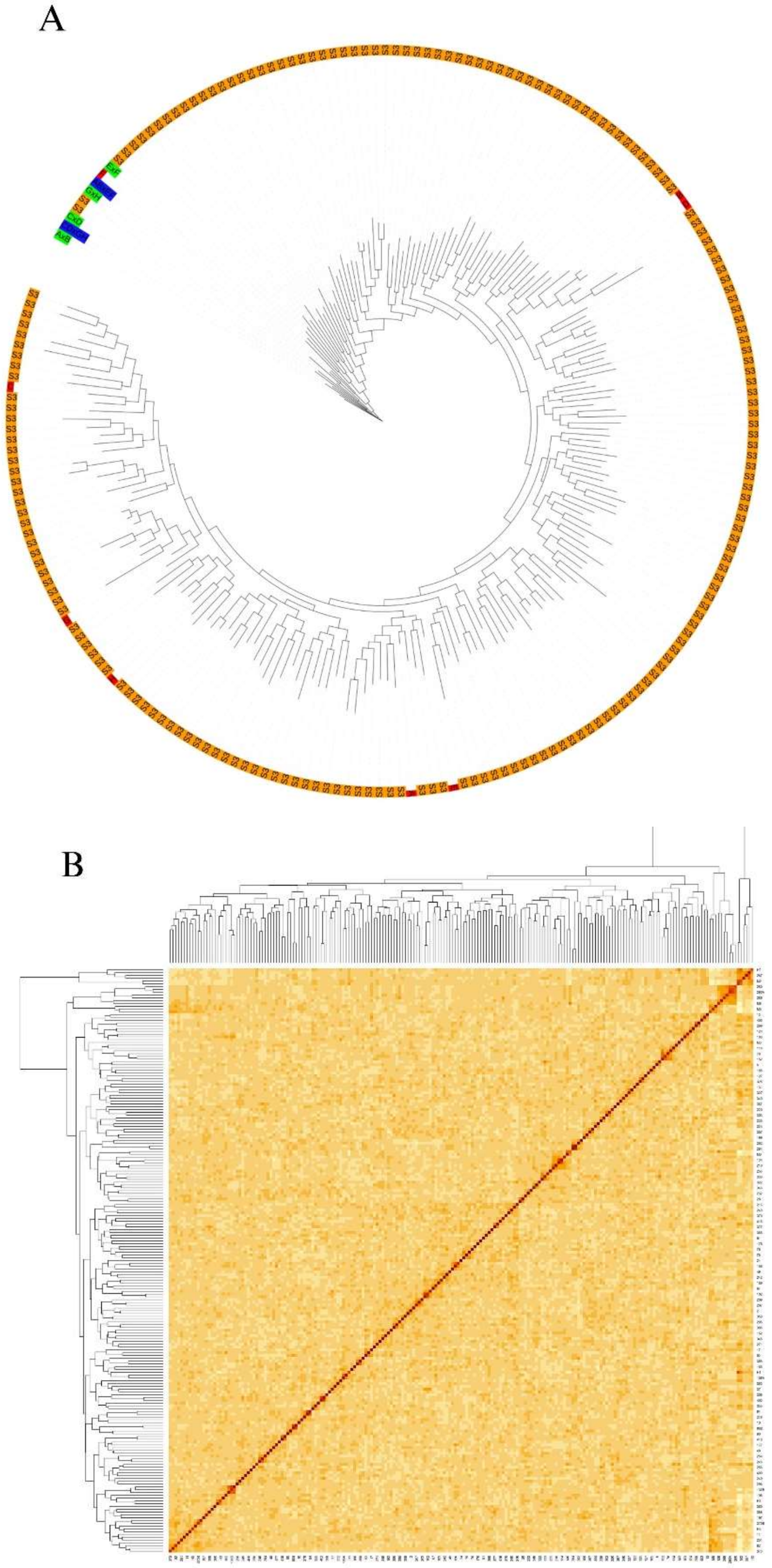

Supplementary figure 1. Population structure analysis for MAGIC population, including founder lines in red colour A: California wonder (*Capsicum annuum*), B: Ecu-994 (*C. chinense*), C: Chile Serrano (*C. annuum*), D: Ají dulce (*C. chinense*), E: Bola (*C. annuum*), F: Pasilla Bajío (*C. annuum*), G: Serrano Criollo de Morelos (*C. annuum*), H: Piquillo (*C. annuum*) (red), F_1 hybrids (green), $F_1 \times F_1$ hybrids (blue) and the 200 S3-individuals (orange) in A: maximum likelihood phylogenetic tree and B: kinship matrix.
