# Supplementary material for: First interspecific multi-parent advanced generation inter-cross (MAGIC) population in Capsicum peppers: development, phenotypic evaluation, genomic analysis, and prospects
Source: Hortic Res. 2025 Jul 16;12(10):uhaf182. doi: 10.1093/hr/uhaf182 (PMC12537016; doi:10.1093/hr/uhaf182)
Supplement: Web_Material_uhaf182 [file web_material_uhaf182.zip › Supplementary Figure 2.pdf]

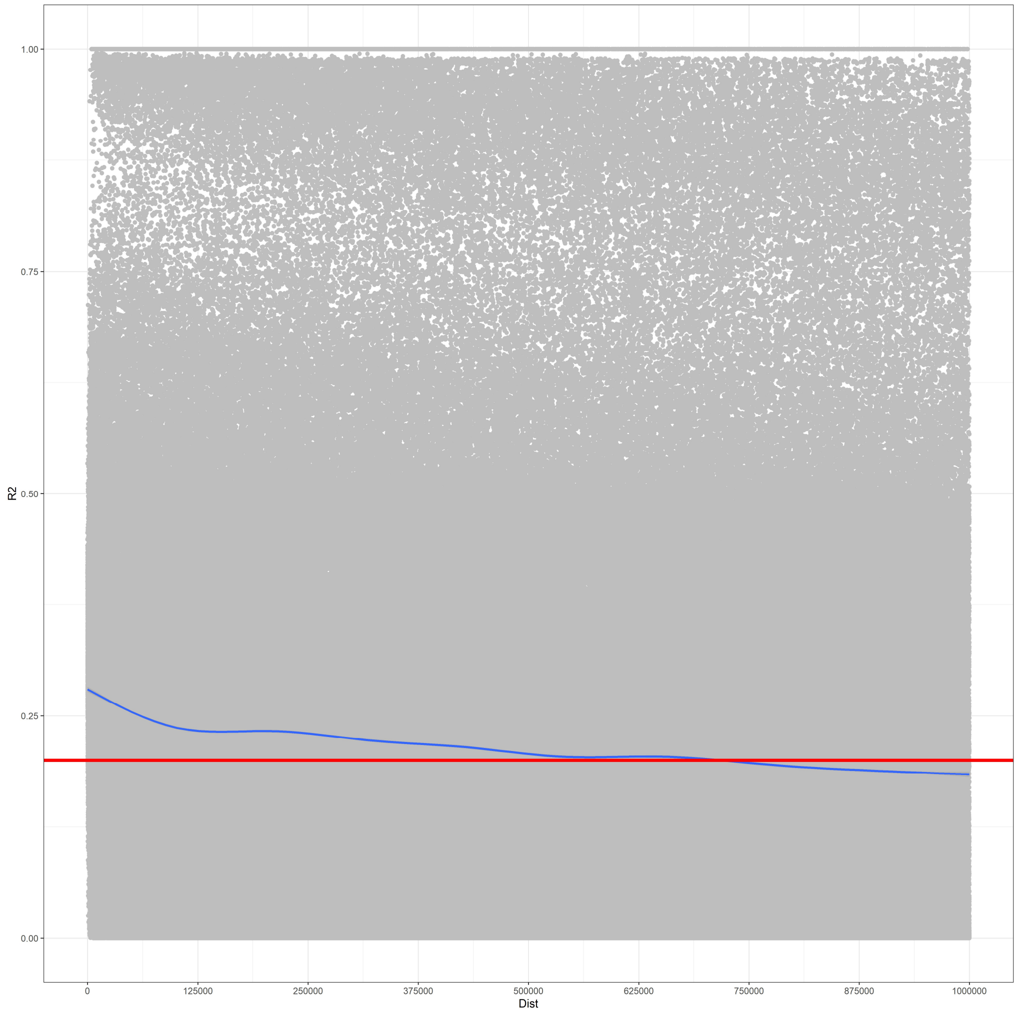

Supplementary figure 2. LD decay (r^2) in Kb off all SNPs pairs related to the physical distance of pairs of SNPs.
