# Supplementary material for: First interspecific multi-parent advanced generation inter-cross (MAGIC) population in Capsicum peppers: development, phenotypic evaluation, genomic analysis, and prospects
Source: Hortic Res. 2025 Jul 16;12(10):uhaf182. doi: 10.1093/hr/uhaf182 (PMC12537016; doi:10.1093/hr/uhaf182)

Supplementary Figure 3. QQ plots and Manhattan plots built with the BLINK and MLMM statistical models for stem colour, nodal anthocyanin, stem pubescence, filament colour, anthocyanin stripes, fruit colour at intermediate stage, fruit colour at mature stage, pedicel persistence with the fruit, and fruit wall consistency. Horizontal lines in the Manhattan plots represent FDR significance threshold at p = 0.05.

STEM COLOUR. BLINK

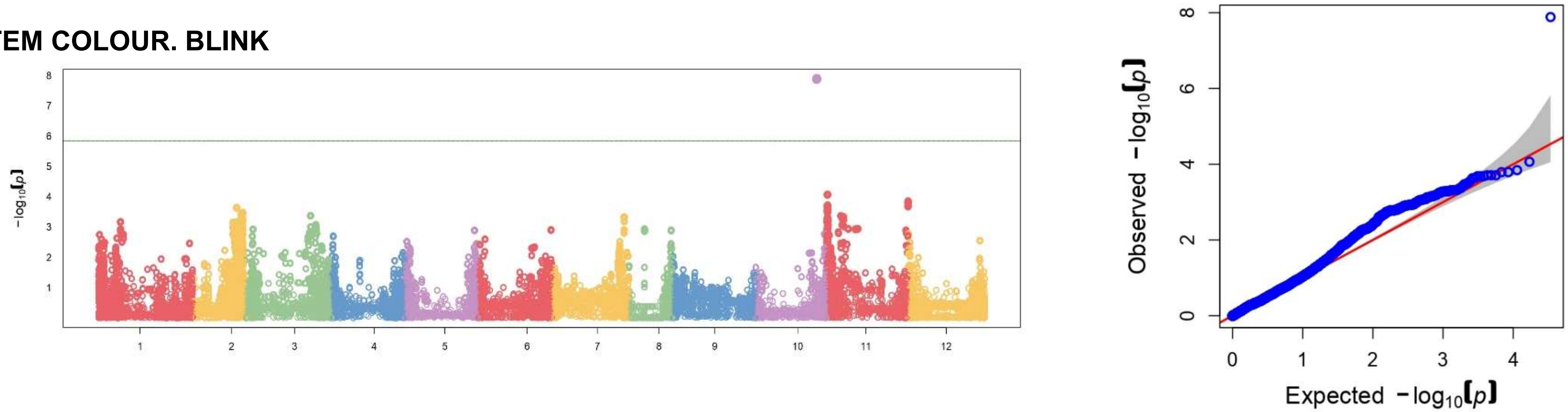

NODAL ANTHOCYANIN. BLINK

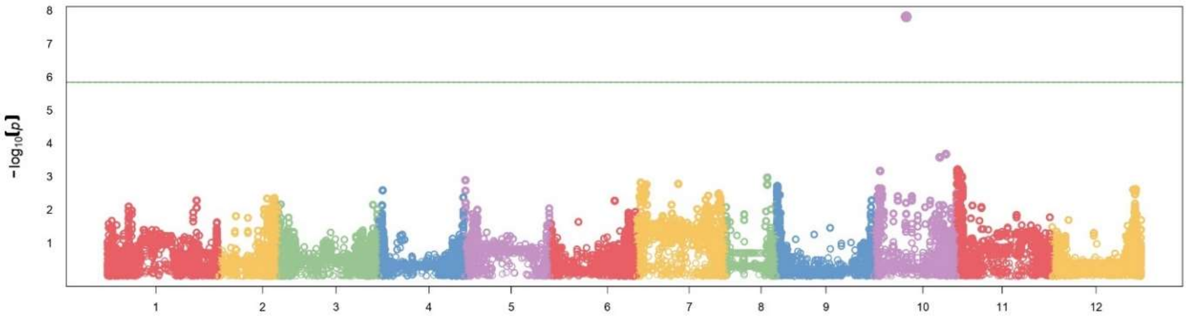

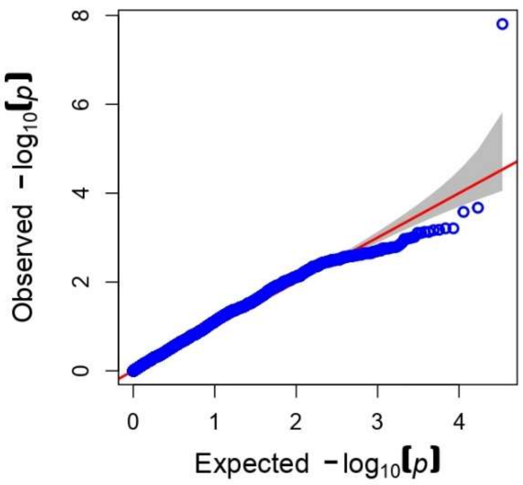

NODAL ANTHOCYANIN. MLMM

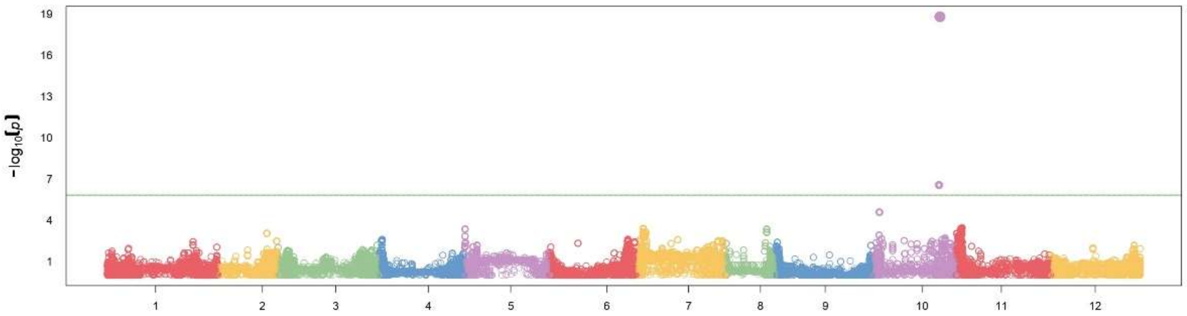

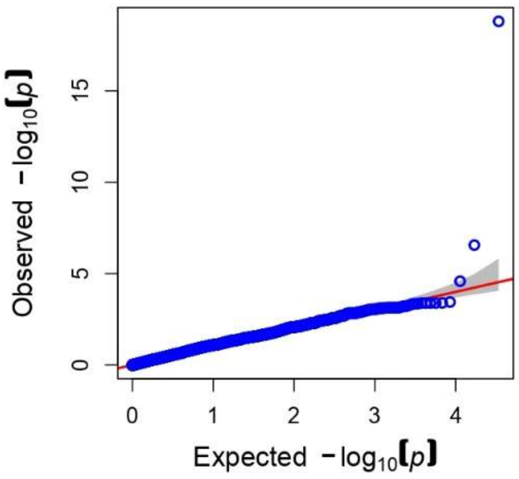

STEM PUBESCENCE. BLINK

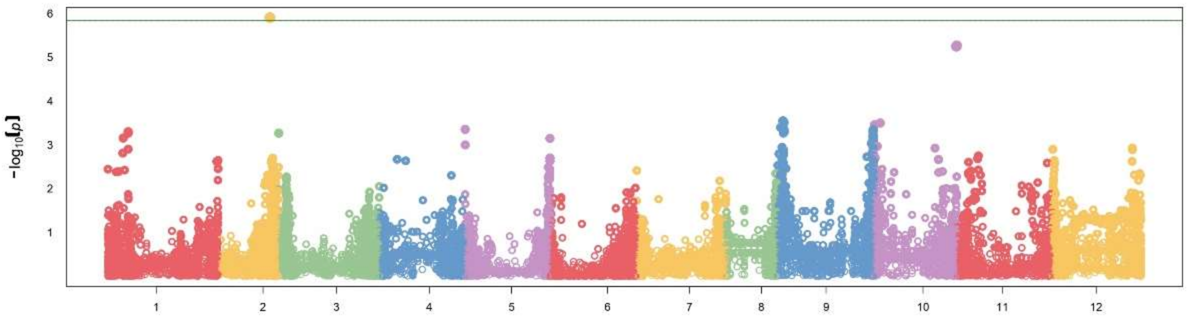

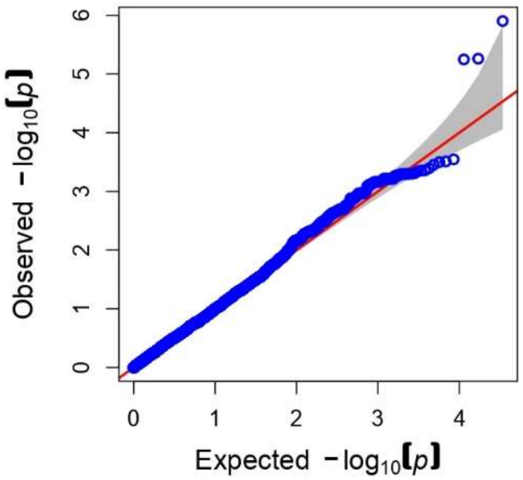

STEM PUBESCENCE. MLMM

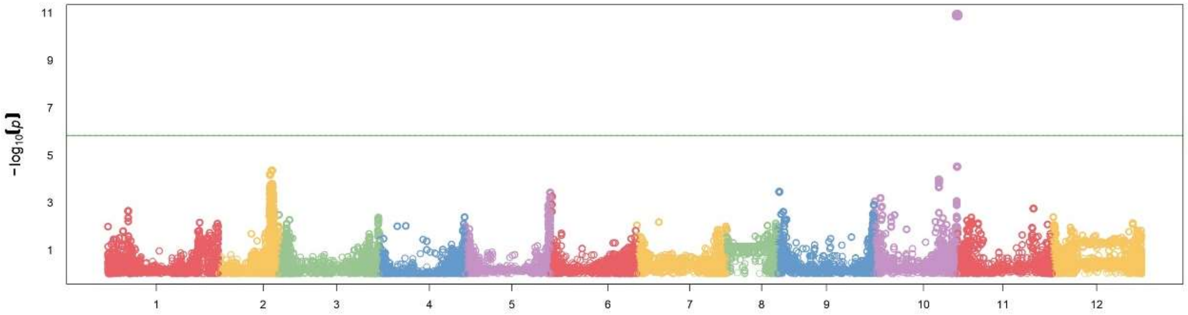

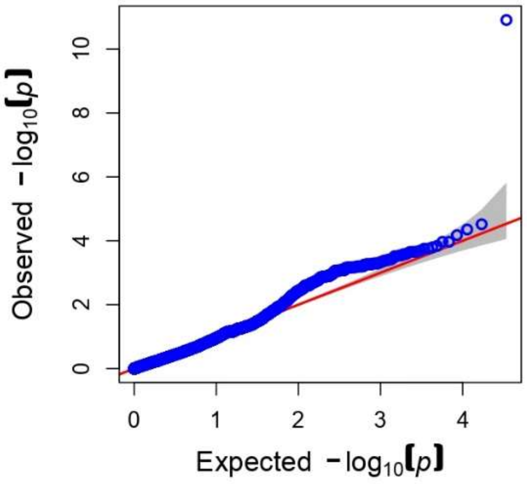

FILAMENT COLOUR. MLMM

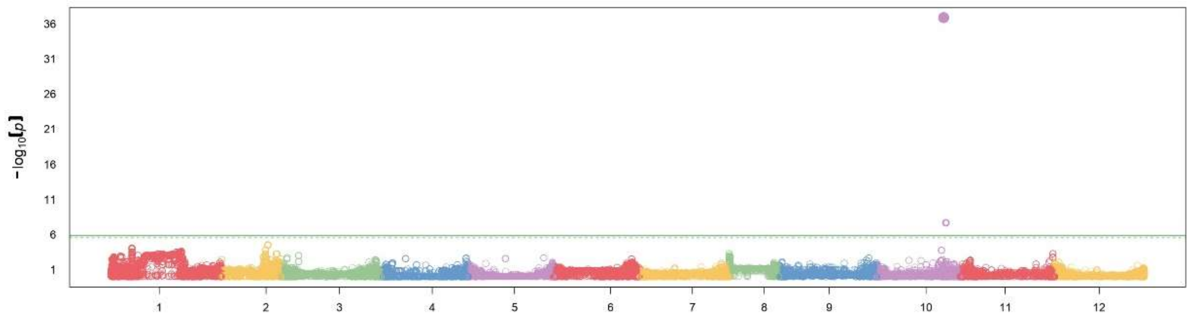

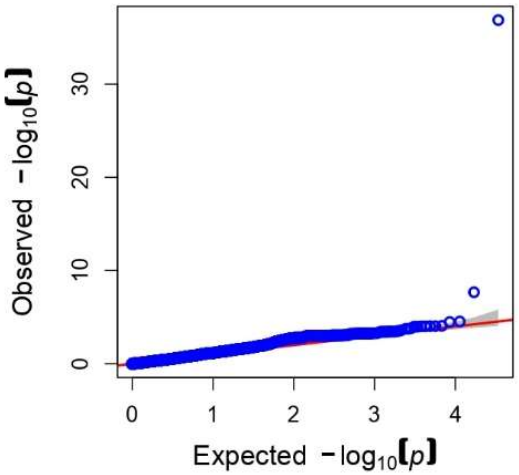

ANTHOCYANIN STRIPES. BLINK

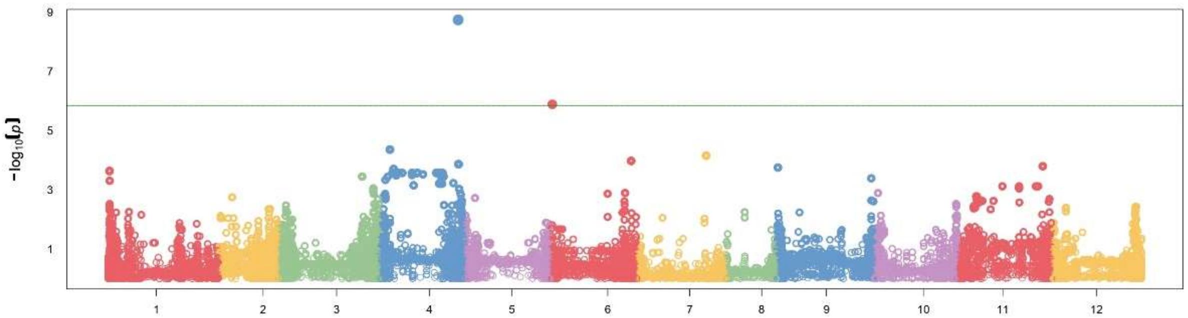

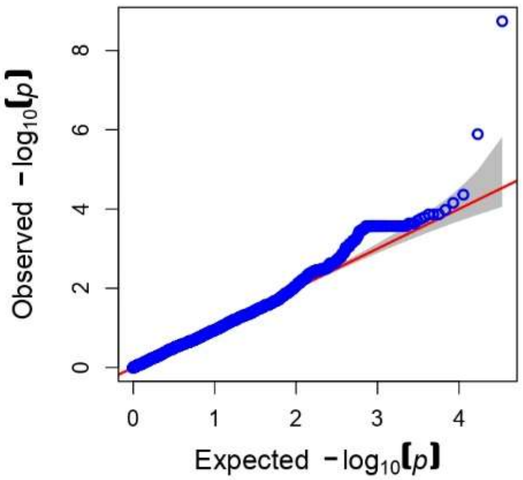

FRUIT COLOUR AT INTERMEDIATE STAGE. BLINK

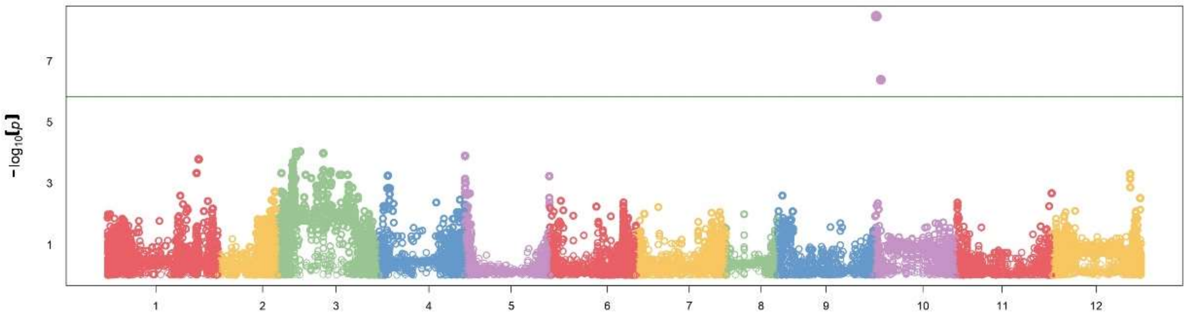

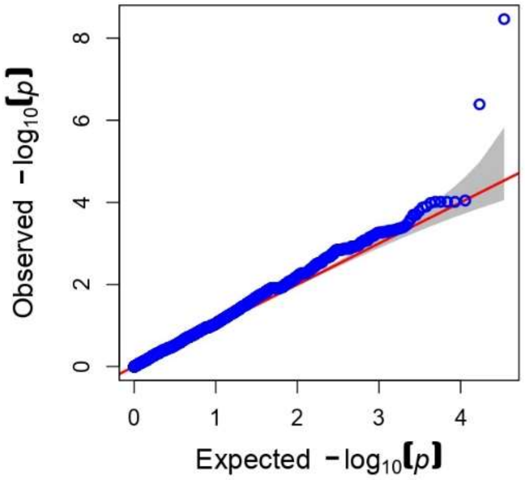

FRUIT COLOUR AT INTERMEDIATE STAGE. MLMM

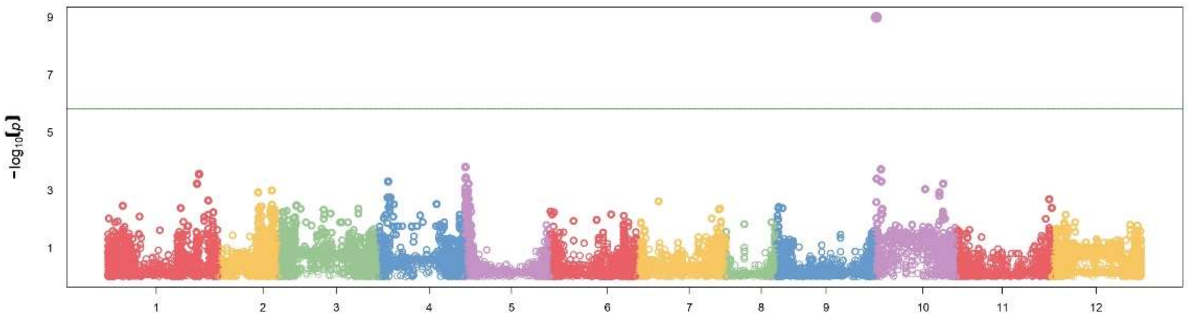

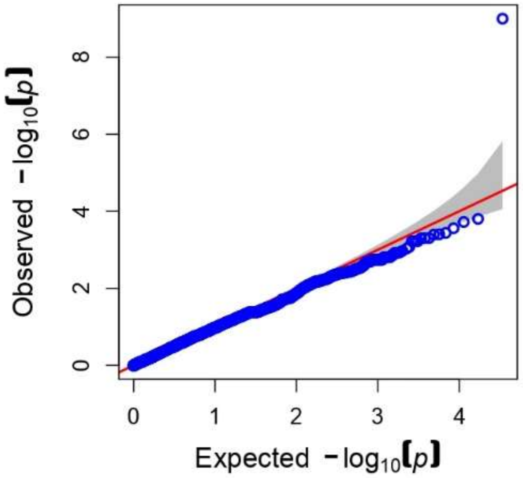

FRUIT COLOUR AT MATURE STAGE. BLINK

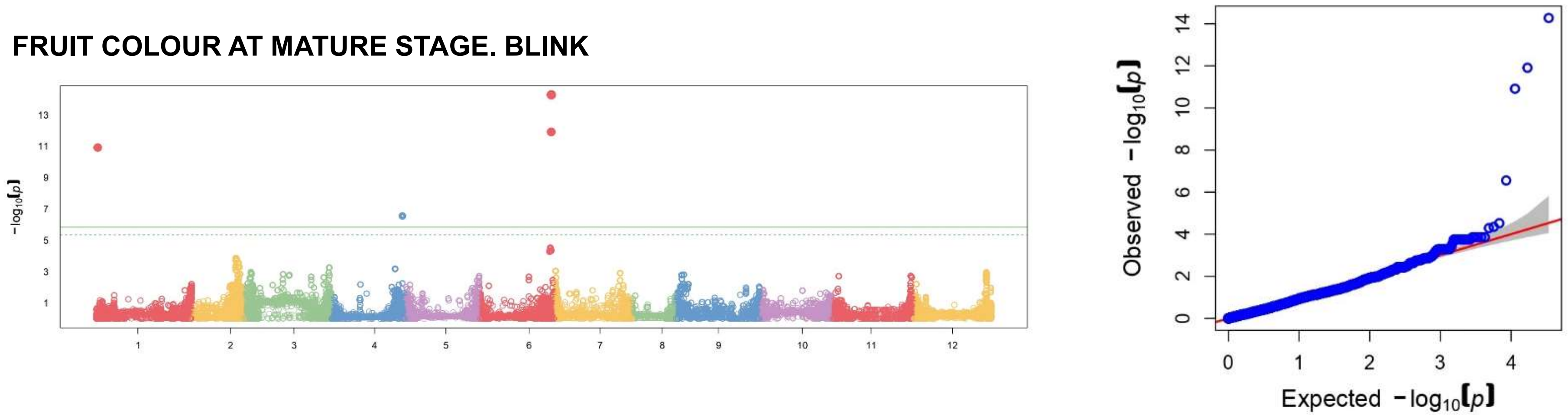

FRUIT COLOUR AT MATURE STAGE. MLMM

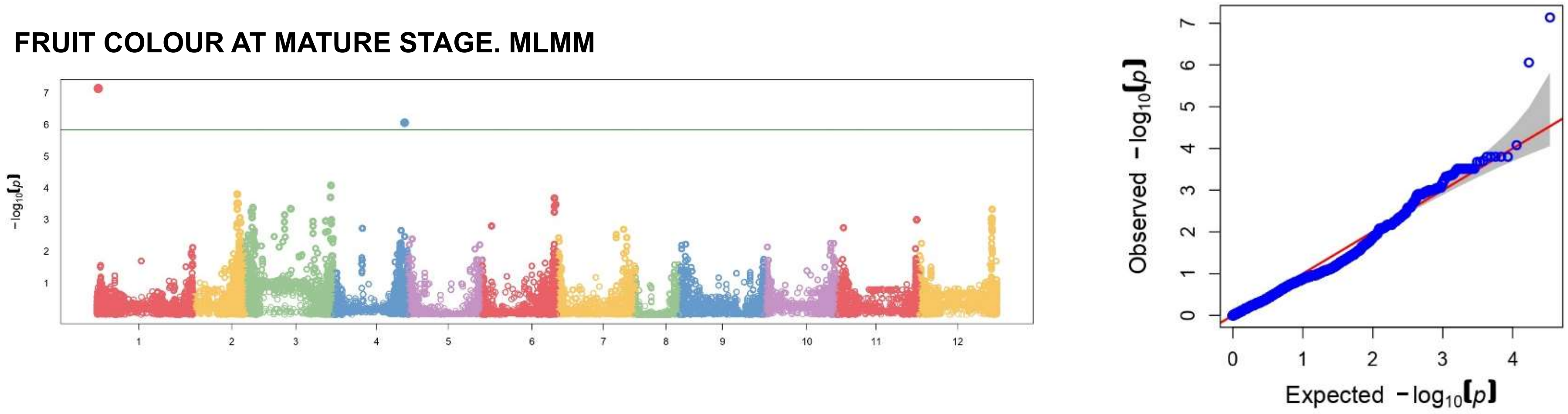

PEDICEL PERSISTENCE WITH THE FRUIT. BLINK

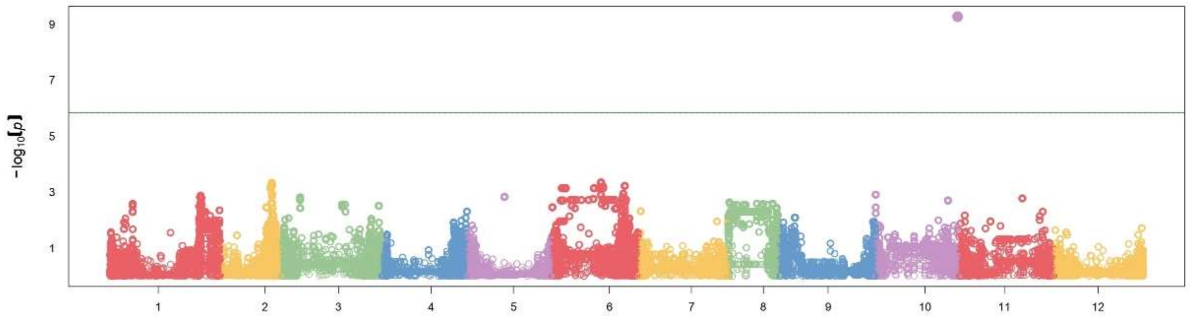

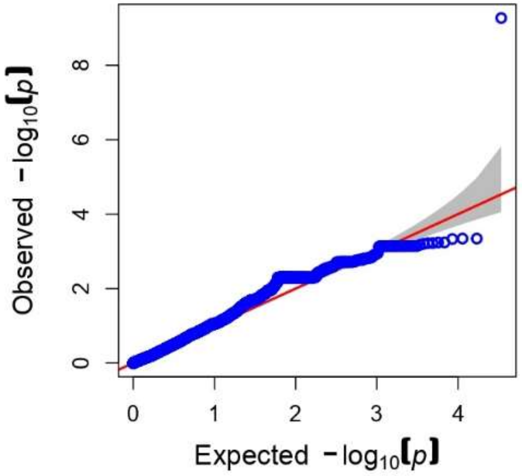

PEDICEL PERSISTENCE WITH THE FRUIT. MLMM

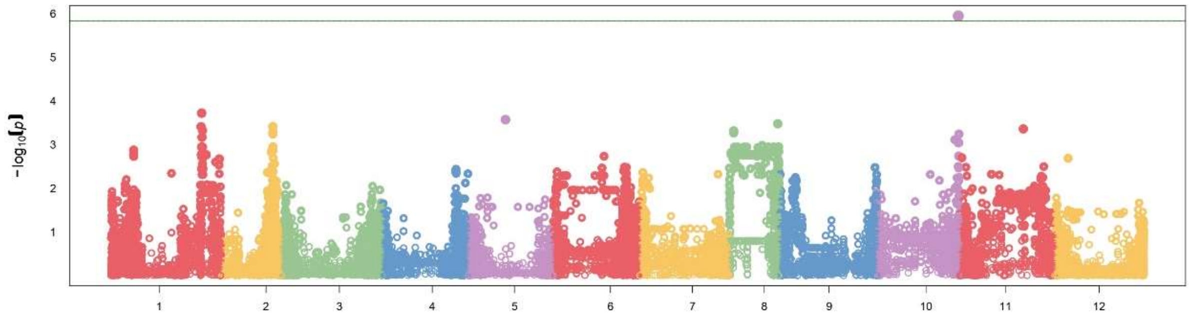

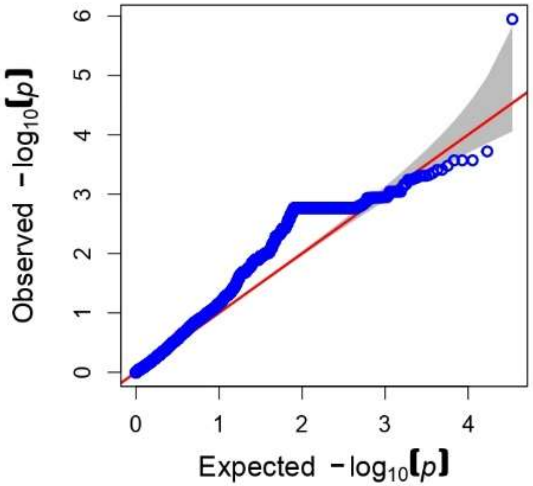

FRUIT WALL CONSISTENCY. BLINK

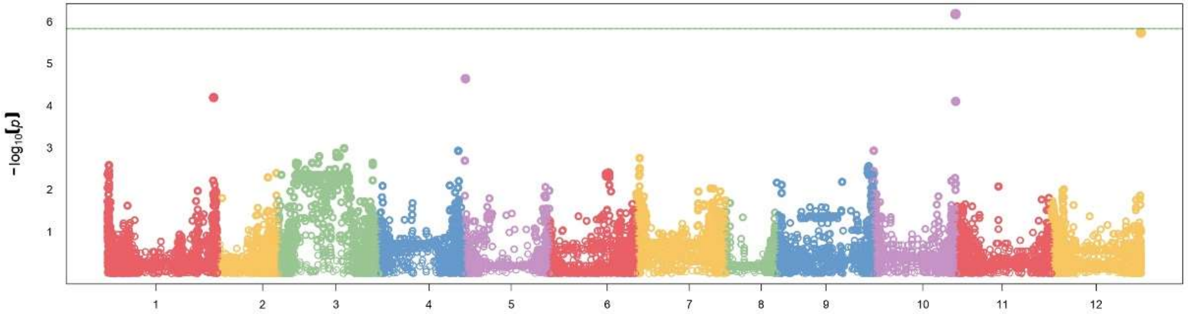

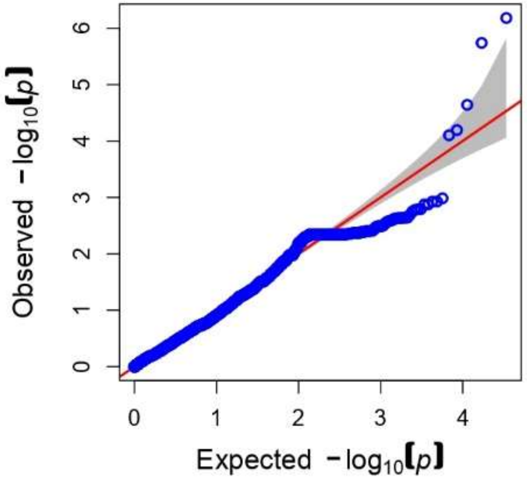

FRUIT WALL CONSISTENCY. MLMM

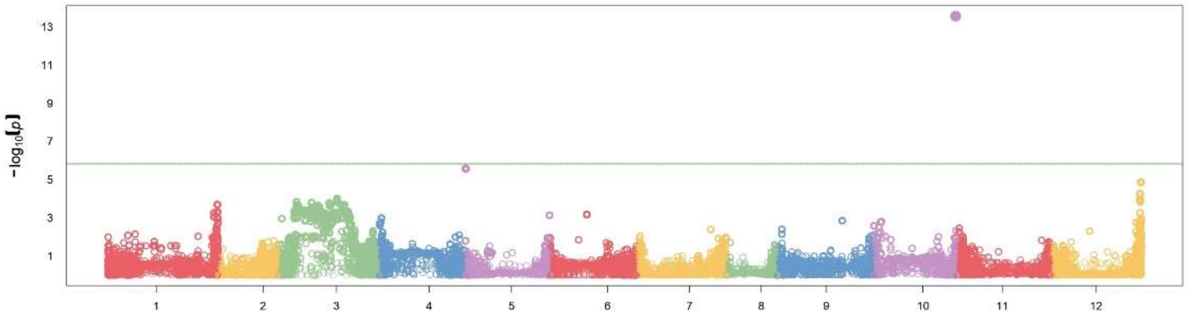

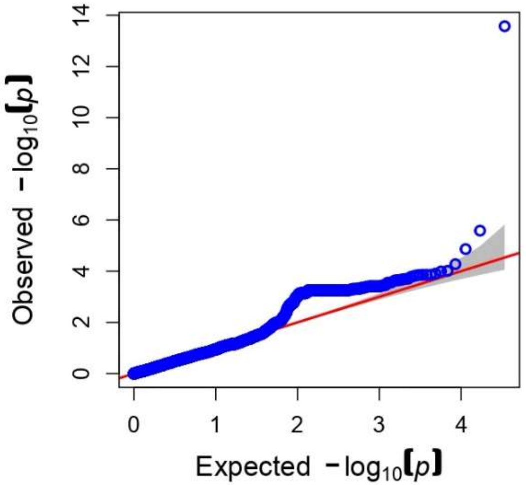

Supplement: Web_Material_uhaf182 [file web_material_uhaf182.zip › Supplementary Figure 3.pdf]
