# Supplementary material for: First interspecific multi-parent advanced generation inter-cross (MAGIC) population in Capsicum peppers: development, phenotypic evaluation, genomic analysis, and prospects
Source: Hortic Res. 2025 Jul 16;12(10):uhaf182. doi: 10.1093/hr/uhaf182 (PMC12537016; doi:10.1093/hr/uhaf182)
Supplement: Web_Material_uhaf182 [file web_material_uhaf182.zip › Supplementary Table 1.docx]

Supplementary table 1. Qualitative trait values for founder lines A: California wonder (*Capsicum annuum*), B: Ají Dulce (*C. chinense*), C: Chile Serrano (*C. annuum*), D: Ecu-994 (*C. chinense*), E: Bola (*C. annuum*), F: Serrano Criollo de Morelos (*C. annuum*), G: Piquillo (*C. annuum*), H: Pasilla Bajío (*C. annuum*), F_1_ and F_1_ × F_1_ hybrids; and phenotype distribution observed in the S3 and S4 progenies.

|  | **Founder lines** | | | | | | |  |
| --- | --- | --- | --- | --- | --- | --- | --- | --- |
| **Trait abbreviation** | **A** | **B** | **C** | **D** | **E** | **F** | **G** | **H** |
| **SC^1^** | Green | Green | Green | Green | Green | Green | Green | Green |
| **AN** | Light purple | Green | Light purple | Green | Purple | Light purple | Purple | Light purple |
| **SP** | Sparce | Sparce | Intermediate | Sparce | Sparce | Dense | Sparce | Sparce |
| **LD** | Dense | Intermediate | Intermediate | Dense | Intermediate | Dense | Sparce | Sparce |
| **LC** | Light green | Light green | Dark green | Green | Green | Green | Green | Green |
| **LS** | Deltoid | Ovate | Lanceolate | Lanceolate | Ovate | Lanceolate | Ovate | Lanceolate |
| **NFA** | 1 | 2 | 1 | 2 | 1 | 1 | 1 | 1 |
| **FP** | Pendant | Intermediate | Pendant | Intermediate | Pendant | Erect | Intermediate | Pendant |
| **AC** | Pale blue | Purple | Purple | Purple | Blue | Pale blue | Pale blue | Purple |
| **FC** | White | Purple | White | Purple | White | White | White | White |
| **SE** | Inserted | Exserted | Exserted | Same level | Inserted | Exserted | Same level | Exserted |
| **CM** | Intermediate | Enture | Dentate | Enture | Intermediate | Dentate | Enture | Dentate |
| **CAC** | Present | Present | Absent | Present | Absent | Absent | Present | Absent |
| **AS** | Absent | Absent | Absent | Absent | Absent | Absent | Absent | Absent |
| **FCI** | Light green | Green | Dark green | Light green | Green | Light green | Green | Dark green |
| **FCM** | Lemon-yellow | Light red | Red | Red | Red | Red | Dark red | Brown |
| **FS** | Blocky | Disc | Elongated | Triangular | Blocky | Elongated | Triangular | Elongated |
| **FSPA** | Lobate | Cordate | Acute | Cordate | Cordate | Cordate | Lobate | Acute |
| **NB** | Absent | Absent | Present | Absent | Absent | Absent | Absent | Absent |
| **FSBE** | Sunken | Blunt | Blunt | Pointed | Blunt | Pointed | Sunken | Pointed |
| **FSC** | Intermediate | Slightly corrugated | Slightly corrugated | Slightly corrugated | Slightly corrugated | Slightly corrugated | Slightly corrugated | Intermediate |
| **PP** | Persistent | Slight | Slight | Intermediate | Persistent | Intermediate | Persistent | Intermediate |
| **FWC** | Present | Present | Absent | Present | Present | Present | Present | Present |
|  |  |  |  |  |  |  |  |  |
|  | **Offsprings** | | | | | | | |
| **Trait abbreviation** | **A × B** | **C × D** | **E × F** | **G × H** | **AB × EF** | **CD × GH** | **S3 progeny** | **S4 progeny** |
| **SC** | Green | Green | Green | Green | Green | Green | Green (83%) | Green (89%) |
|  |  |  |  |  |  |  | Green with purple stripes (12%) | Green with purple stripes (7%) |
|  |  |  |  |  |  |  | Purple (5%) | Purple (4%) |
| **AN** | Green | Purple | Light purple | Light purple | Green | Green | Green (26%) | Green (32%) |
|  |  |  |  |  |  |  | Light purple (32%) | Light purple (26%) |
|  |  |  |  |  |  |  | Purple (41%) | Purple (42%) |
| **SP** | Sparce | Intermediate | Intermediate | Sparce | Sparce | Sparce | Sparce (74%) | Sparce (67%) |
|  |  |  |  |  |  |  | Intermediate (23%) | Intermediate (26%) |
|  |  |  |  |  |  |  | Dense (3%) | Dense (7%) |
| **LD** | Intermediate | Intermediate | Dense | Intermediate | Intermediate | Intermediate | Sparce (17%) | Sparce (20%) |
|  |  |  |  |  |  |  | Intermediate (55%) | Intermediate (59%) |
|  |  |  |  |  |  |  | Dense (27%) | Dense (21%) |
| **LC** | Light green | Green | Green | Dark green | Green | Dark green | Light green (18%) | Light green (14%) |
|  |  |  |  |  |  |  | Green (62%) | Green (65%) |
|  |  |  |  |  |  |  | Dark green (19%) | Dark green (21%) |
| **LS** | Lanceolate | Lanceolate | Lanceolate | Ovate | Ovate | Ovate | Deltoid (17%) | Deltoid (14%) |
|  |  |  |  |  |  |  | Ovate (41%) | Ovate (58%) |
|  |  |  |  |  |  |  | Lanceolate (42%) | Lanceolate (28%) |
| **NFA** | 1 | 1 | 1 | 1 | 1 | 1 | 1 (80%) | 1 (85%) |
|  |  |  |  |  |  |  | 2 (20%) | 2 (15%) |
| **FP** | Erect | Intermediate | Pendant | Pendant | Intermediate | Pendant | Pendant (35%) | Pendant (27%) |
|  |  |  |  |  |  |  | Intermediate (50%) | Intermediate (43%) |
|  |  |  |  |  |  |  | Erect (15%) | Erect (20%) |
| **AC** | Purple | Purple | Blue | Pale blue | Pale blue | Purple | Pale blue (25%) | Pale blue (22%) |
|  |  |  |  |  |  |  | Blue (34%) | Blue (48%) |
|  |  |  |  |  |  |  | Purple (41%) | Purple (30%) |
| **FC** | Purple | Purple | White | White | White | Light purple | White (80%) | White (80%) |
|  |  |  |  |  |  |  | Yellow (1%) | Yellow (0%) |
|  |  |  |  |  |  |  | Blue (1%) | Blue (1%) |
|  |  |  |  |  |  |  | Light purple (6%) | Light purple (9%) |
|  |  |  |  |  |  |  | Purple (13%) | Purple (10%) |
| **SE** | Exserted | Exserted | Same level | Exserted | Same level | Exserted | Inserted (5%) | Inserted (4%) |
|  |  |  |  |  |  |  | Same level (30%) | Same level (22%) |
|  |  |  |  |  |  |  | Exserted (66%) | Exserted (72%) |
| **CM** | Intermediate | Intermediate | Intermediate | Dentate | Intermediate | Intermediate | Enture (21%) | Enture (12%) |
|  |  |  |  |  |  |  | Intermediate (69%) | Intermediate (73%) |
|  |  |  |  |  |  |  | Dentate (10%) | Dentate (15%) |
| **CAC** | Absent | Present | Absent | Present | Present | Present | Absent (78%) | Absent (82%) |
|  |  |  |  |  |  |  | Present (22%) | Present (18%) |
| **AS** | Absent | Absent | Absent | Absent | Absent | Absent | Absent (98%) | Absent (95%) |
|  |  |  |  |  |  |  | Present (2%) | Present (5%) |
| **FCI** | Green | Green | Light green | Dark green | Light green | Light green | Light green (13%) | Light green (15%) |
|  |  |  |  |  |  |  | Green (59%) | Green (58%) |
|  |  |  |  |  |  |  | Dark green (28%) | Dark green (27%) |
| **FCM** | Red | Red | Red | Dark red | Red | Red | Lemon-yellow (1%) | Lemon-yellow (1%) |
|  |  |  |  |  |  |  | Pale orange-yellow (2%) | Pale orange-yellow (3%) |
|  |  |  |  |  |  |  | Orange-yellow (3%) | Orange-yellow (2%) |
|  |  |  |  |  |  |  | Pale orange (2%) | Pale orange (1%) |
|  |  |  |  |  |  |  | Light red (5%) | Light red (5%) |
|  |  |  |  |  |  |  | Red (63%) | Red (70%) |
|  |  |  |  |  |  |  | Dark red (16%) | Dark red (12%) |
|  |  |  |  |  |  |  | Purple (1%) | Purple (0%) |
|  |  |  |  |  |  |  | Brown (3%) | Brown (4%) |
|  |  |  |  |  |  |  | Black (3%) | Black (2%) |
| **FS** | Triangular | Triangular | Triangular | Triangular | Elongate | Blocky | Elongate (20%) | Elongate (17%) |
|  |  |  |  |  |  |  | Almost round (8%) | Almost round (11%) |
|  |  |  |  |  |  |  | Triangular (27%) | Triangular (40%) |
|  |  |  |  |  |  |  | Blocky (46%) | Blocky (32%) |
| **FSPA** | Truncate | Acute | Cordate | Cordate | Cordate | Cordate | Acute (17%) | Acute (23%) |
|  |  |  |  |  |  |  | Truncate (25%) | Truncate (31%) |
|  |  |  |  |  |  |  | Cordate (51%) | Cordate (41%) |
|  |  |  |  |  |  |  | Lobate (7%) | Lobate (5%) |
| **NB** | Absent | Absent | Absent | Absent | Absent | Absent | Absent (99%) | Absent (99%) |
|  |  |  |  |  |  |  | Present (1%) | Present (1%) |
| **FSBE** | Blunt (50%) | Sunken | Blunt | Pointed | Blunt | Sunken | Pointed (12%) | Pointed (12%) |
|  | Sunken (50%) |  |  |  |  |  | Blunt (43%) | Blunt (32%) |
|  |  |  |  |  |  |  | Sunken (45%) | Sunken (56%) |
| **FSC** | Slightly corrug (70%) | Slightly corrugated | Slightly corrugated | Slightly corrug (80%) | Slightly corrug (20%) | Slightly corrug (60%) | Slightly corrugated (63%) | Slightly corrugated (57%) |
|  | Intermediate (20%) |  |  | Intermediate (20%) | Intermediate (50%) | Intermediate (40%) | Intermediate (27%) | Intermediate (37%) |
|  | Corrugated (10%) |  |  |  | Corrugated (30%) |  | Corrugated (10%) | Corrugated (10%) |
| **PP** | Intermediate | Intermediate | Persistent | Persistent | Intermediate | Slight | Slight (20%) | Slight (18%) |
|  |  |  |  |  |  |  | Intermediate (63%) | Intermediate (56%) |
|  |  |  |  |  |  |  | Persistent (16%) | Persistent (32%) |
| **FWC** | Present | Present | Present | Present | Present | Absent | Absent (18%) | Absent (22%) |
|  |  |  |  |  |  |  | Present (82%) | Present (78%) |

^1^ SC: stem colour, AN: nodal anthocyanin, SP: stem pubescence, LD: leaf density, LC: leaf colour, LS: leaf shape, NFA: number of flowers per axil, FP: flower position, AC: anther colour, FC: filament colour, SE: stigma exsertion, CM: calyx margin, CAC: calyx annular constriction, AS: anthocyaninic spots, FCI: fruit colour intermediate, FCM: fruit colour mature, FS: fruit shape, FSPA: fruit shape pedicel attachment, NB: neck at fruit base, FSBE: fruit shape at blossom end, FSC: , fruit cross-section corrugation, PP: pedicel persistence, FWC: fruit wall consistency.
