# Supplementary material for: First interspecific multi-parent advanced generation inter-cross (MAGIC) population in Capsicum peppers: development, phenotypic evaluation, genomic analysis, and prospects
Source: Hortic Res. 2025 Jul 16;12(10):uhaf182. doi: 10.1093/hr/uhaf182 (PMC12537016; doi:10.1093/hr/uhaf182)
Supplement: Web_Material_uhaf182 [file web_material_uhaf182.zip › Supplementary Table 2.docx]

Supplementary table 2. Quantitative trait values (mean ± standard deviation) for founder lines A: California wonder (*Capsicum annuum*), B: Ají Dulce (*C. chinense*), C: Chile Serrano (*C. annuum*), D: Ecu-994 (*C. chinense*), E: Bola (*C. annuum*), F: Serrano Criollo de Morelos (*C. annuum*), G: Piquillo (*C. annuum*), H: Pasilla Bajío (*C. annuum*), F_1_, F_1_ × F_1_ hybrids, and the S3 and S4 progenies; and coefficient of variation (CV %) for the later generations.

|  | **Founder lines** | | | | | | | | | | | | | | | | |  |
| --- | --- | --- | --- | --- | --- | --- | --- | --- | --- | --- | --- | --- | --- | --- | --- | --- | --- | --- |
| **Trait abbreviation** | | **A** | | **B** | | **C** | | **D** | | **E** | | **F** | | **G** | | **H** | |  |
| **SL^1^** | | 238.3 ± 25 | | 570 ± 72 | | 853.3 ± 100 | | 380 ± 35 | | 441.7 ± 7 | | 272.5 ± 60 | | 275 ± 47 | | 395 ± 78 | |  |
| **ILe** | | 80 ± 21 | | 138.8 ± 19 | | 100 ± 21 | | 95 ± 3 | | 68.8 ± 5 | | 103.8 ± 33 | | 92.5 ± 10 | | 100 ± 28 | |  |
| **IN** | | 12.5 ± 0.7 | | 9.5 ± 0.7 | | 17.5 ± 6 | | 15.5 ± 5 | | 13 ± 1 | | 13 ± 2 | | 10 ± 1 | | 11.5 ± 2 | |  |
| **LLe** | | 158.5 ± 19 | | 134.3 ± 10 | | 81.5 ± 9 | | 77 ± 5 | | 134.3 ± 14 | | 119.8 ± 13 | | 121.5 ± 9 | | 122.5 ± 8 | |  |
| **LWi** | | 79 ± 6 | | 75.5 ± 7 | | 32 ± 3 | | 45.7 ± 4 | | 76.3 ± 6 | | 38 ± 3 | | 67 ± 5 | | 57.3 ± 7 | |  |
| **LL** | | 37.5 ± 1 | | 38.9 ± 1 | | 32.9 ± 2 | | 34.5 ± 1 | | 34.4 ± 0.5 | | 32.1 ± 0.5 | | 33.5 ± 1 | | 30.6 ± 1 | |  |
| **LA** | | -14.7 ± 1 | | -16.3 ± 1 | | -10.2 ± 1 | | -12.5 ± 0.4 | | -11.8 ± 1 | | -10.4 ± 1 | | -11.6 ± 1 | | -8.8 ± 2 | |  |
| **LB** | | 17.3 ± 1 | | 21.5 ± 1 | | 13.1 ± 2 | | 14.1 ± 0.5 | | 14.8 ± 1 | | 13.9 ± 1 | | 15.5 ± 2 | | 11.5 ± 2 | |  |
| **FL** | | 61.2 ± 2 | | 42.4 ± 2 | | 41.5 ± 2 | | 41.1 ± 2 | | 36.5 ± 1 | | 39.8 ± 2 | | 35.2 ± 2 | | 27.7 ± 1 | |  |
| **FA** | | 0.5 ± 3 | | 38.4 ± 2 | | 40.3 ± 1 | | 39.9 ± 2 | | 35.5 ± 2 | | 38.8 ± 3 | | 32.7 ± 5 | | 3.1 ± 1 | |  |
| **FB** | | 56 ± 3 | | 27.3 ± 3 | | 25.9 ± 4 | | 26.9 ± 3 | | 18.3 ± 2 | | 23.5 ± 3 | | 15.9 ± 3 | | 3.5 ± 1 | |  |
| **FLe** | | 71.3 ± 9 | | 15.3 ± 2 | | 36 ± 4 | | 55.2 ± 3 | | 32.5 ± 4 | | 96.4 ± 11 | | 77.1 ± 9 | | 132.4 ± 33 | |  |
| **FWi** | | 63.6 ± 2 | | 26.3 ± 4 | | 12.6 ± 1 | | 16.6 ± 3 | | 26.4 ± 2 | | 19.7 ± 2 | | 38.7 ± 5 | | 26.3 ± 4 | |  |
| **FW** | | 124 ± 20 | | 3.8 ± 1 | | 3.2 ± 1 | | 5.9 ± 1 | | 12.4 ± 4 | | 15.4 ± 4 | | 39.1 ± 12 | | 20.4 ± 11 | |  |
| **PL** | | 41 ± 5 | | 29 ± 3 | | 35 ± 05 | | 27 ± 3 | | 34 ± 5 | | 47 ± 10 | | 55 ± 10 | | 59 ± 5 | |  |
| **PFB** | | 0.9 ± 0.1 | | 0.9 ± 0.1 | | 0.8 ± 0.1 | | 1.3 ± 0.2 | | 1.2 ± 0.1 | | 1.1 ± 0.1 | | 1 ± 0.1 | | 0.9 ± 0.1 | |  |
| **DFB** | | 0.8 ± 0.1 | | 0.7 ± 0.1 | | 0.8 ± 0.1 | | 0.5 ± 0.01 | | 0.9 ± 0.01 | | 0.7 ± 0.1 | | 0.7 ± 0.1 | | 0.7 ± 0.1 | |  |
| **FST** | | 1.3 ± 0.2 | | 1.5 ± 0.1 | | 1.1 ± 0.2 | | 2.5 ± 0.4 | | 1.4 ± 0.1 | | 1.6 ± 0.2 | | 1.4 ± 0.4 | | 1.3 ± 0.1 | |  |
| **NL** | | 3.4 ± 0.5 | | 2.5 ± 0.5 | | 2.3 ± 0.5 | | 2.9 ± 0.3 | | 2.7 ± 0.5 | | 2.3 ± 0.5 | | 2.5 ± 0.7 | | 2.2 ± 0.4 | |  |
| **PMV** | | 0.76 ± 0.05 | | 0.41 ± 0.01 | | 0.59 ± 0.01 | | 0.31 ± 0.01 | | 0.69 ± 0.01 | | 0.68 ± 0.04 | | 0.78 ± 0.03 | | 1.3 ± 0.01 | |  |
| **PBV** | | 93.9 ± 1 | | 94.1 ± 2 | | 65.2 ± 9 | | 22.7 ± 14 | | 90.5 ± 7 | | 90.8 ± 5 | | 76.2 ± 17 | | 88.1 ± 2 | |  |
| **PGV** | | 93.8 ± 1 | | 94.1 ± 2 | | 64.2 ± 8 | | 22.5 ± 13 | | 90.4 ± 8 | | 88.2 ± 6 | | 76.2 ± 16 | | 88.1 ± 2 | |  |
| **SW** | | 23.7 ± 5 | | 29.3 ± 8 | | 55.4 ± 18 | | 9.6 ± 13 | | 43.8 ± 5 | | 40.9 ± 12 | | 9 ± 3 | | 15.3 ± 7 | |  |
|  |  | |  | |  | |  | |  |  |  | |  | |  | |  | |
|  | **Offsprings** | | | | | | | | | | | | | | | | | |
| **Trait abbreviation** | **A × B** | | **C × D** | | **E × F** | | **G × H** | | **AB × EF** | **CD × GH** | **S3 progeny** | | | | **S4 progeny** | | | |
|  |  | |  | |  | |  | |  |  | **Mean** | | **CV** | | **Mean** | | **CV** | |
| **SL** | 510 ± 158 | | 820 ± 210 | | 455 ± 87 | | 410 ± 62 | | 310 ± 42 | 395 ± 96 | 391.7 ± 167.8 | | 42.8 | | 370.2 ± 178.8 | | 48.2 | |
| **ILe** | 82.5 ± 25 | | 167.5 ± 45 | | 107.5 ± 33 | | 117.5 ± 55 | | 90 ± 23 | 185 ± 44 | 111.2 ± 64.5 | | 57.9 | |  | |  | |
| **IN** | 18 ± 4 | | 5 ± 2 | | 16 ± 5 | | 13 ± 4 | | 15 ± 4 | 12 ± 4 | 15.8 ± 4.7 | | 30.1 | |  | |  | |
| **LLe** | 148.5 ± 6 | | 119.8 ± 4 | | 125.8 ± 8 | | 135.5 ± 6 | | 131 ± 4 | 113.3 ± 12 | 109.1 ± 18.7 | | 17.1 | |  | |  | |
| **LWi** | 73.8 ± 4 | | 59 ± 3 | | 62 ± 4 | | 73.5 ± 4 | | 60.5 ± 5 | 65.5 ± 5 | 56.3 ± 10.9 | | 19.3 | |  | |  | |
| **LL** | 38.7 ± 0.5 | | 33.9 ± 0.5 | | 33.4 ± 0.5 | | 29.3 ± 1 | | 35 ± 1 | 30.6 ± 1 | 33.5 ± 2.9 | | 8.6 | |  | |  | |
| **LA** | -16.1 ± 0.3 | | -12.9 ± 1 | | -11.9 ± 1 | | -7.6 ± 1 | | -13.1 ± 1 | -7.8 ± 1 | -11.5 ± 2.7 | | 23 | |  | |  | |
| **LB** | 20.4 ± 0.5 | | 15.8 ± 1 | | 15.3 ± 1 | | 9.5 ± 2 | | 17.7 ± 1 | 10.2 ± 1 | 14.7 ± 3.7 | | 25.4 | |  | |  | |
| **FL** | 40.2 ± 2 | | 40.6 ± 3 | | 41.8 ± 3 | | 36.7 ± 2 | | 40.9 ± 2 | 40.2 ± 2 | 0.4 ± 7.9 | | 19.7 | | 37.4 ± 5.6 | | 15 | |
| **FA** | 35.8 ± 3 | | 35.8 ± 3 | | 39.2 ± 3 | | 36.5 ± 3 | | 37.5 ± 2 | 40.5 ± 4 | 33.9 ± 11.6 | | 34.2 | | 34.9 ± 8.4 | | 23.8 | |
| **FB** | 22.9 ± 4 | | 27.1 ± 4 | | 28.3 ± 5 | | 19 ± 2 | | 23.4 ± 3 | 26.4 ± 4 | 24.6 ± 13.4 | | 54.6 | | 21.8 ± 9.3 | | 42.6 | |
| **FLe** | 40 ± 8 | | 35.2 ± 3 | | 44.5 ± 11 | | 104.1 ± 19 | | 37.6 ± 5 | 36 ± 4 | 37.5 ± 15.8 | | 42.2 | | 35.8 ± 16.5 | | 46.1 | |
| **FWi** | 24.9 ± 4 | | 12.4 ± 1 | | 21.3 ± 5 | | 26.9 ± 4 | | 23.2 ± 2 | 17 ± 2 | 20.6 ± 6 | | 29 | | 20.3 ± 6.1 | | 29.8 | |
| **FW** | 9.4 ± 4 | | 2.4 ± 0.6 | | 7.7 ± 1 | | 21.9 ± 6 | | 8.8 ± 2 | 4 ± 1 | 7.6 ± 7.7 | | 101.9 | | 6.9 ± 9.1 | | 132.9 | |
| **PL** | 28 ± 4 | | 32 ± 3 | | 34 ± 5 | | 64 ± 9 | | 45 ± 5 | 34 ± 5 | 33 ± 12 | | 36.3 | |  | |  | |
| **PFB** | 1.1 ± 0.1 | | 1.1 ± 0.1 | | 1.2 ± 0.2 | | 1.1 ± 0.1 | | 1.1 ± 0.1 | 1.2 ± 0.1 | 0.9 ± 0.1 | | 11 | |  | |  | |
| **DFB** | 0.8 ± 0.1 | | 0.9 ± 0.1 | | 0.8 ± 0.1 | | 0.8 ± 0.1 | | 0.8 ± 0.1 | 0.9 ± 0.1 | 0.8 ± 0.1 | | 7.9 | |  | |  | |
| **FST** | 1.4 ± 0.1 | | 1.3 ± 0.1 | | 1.6 ± 0.1 | | 1.3 ± 0.1 | | 1.3 ± 0.1 | 1.4 ± 0.1 | 1.2 ± 0.1 | | 11.5 | |  | |  | |
| **NL** | 2.5 ± 0.5 | | 2.9 ± 0.3 | | 2 ± 0 | | 2 ± 0 | | 2.8 ± 0.5 | 2.7 ± 0.5 | 2.6 ± 0.4 | | 17.6 | | 2.7 ± 0.5 | | 18 | |
| **PMV** | 0.4 ± 0.01 | | 0.4 ± 0.01 | | 0.7 ± 0.06 | | 0.9 ± 0.03 | | 0.6 ± 0.02 | 0.6 ± 0.01 | 0.7 ± 0.1 | | 21 | | 0.6 ± 0.2 | | 28.5 | |
| **PBV** | 6.7 ± 1 | | 5.4 ± 0.9 | | 85.2 ± 12 | | 54.2 ± 9 | | 53.4 ± 10 | 1 ± 0.01 | 40.5 ± 26.5 | | 65.5 | | 41.5 ± 28.7 | | 69 | |
| **PGV** | 5.4 ± 1 | | 5.4 ± 0.8 | | 84 ± 21 | | 46.8 ± 13 | | 51.8 ± 10 | 1 ± 0.01 | 33.2 ± 27.5 | | 82.7 | | 38.6 ± 29.5 | | 76.3 | |
| **SW** | 0 ± 0.1 | | 0 ± 0.1 | | 6.3 ± 1 | | 3 ± 0.5 | | 23.7 ± 4 | 0 ± 0.1 | 11.4 ± 14.5 | | 126.8 | | 6.4 ± 10.1 | | 156.2 | |

^1^ SL: stem length (mm), ILe: internode length (mm), IN: internode number, LLe: leaf length (mm), LWi: leaf width (mm), LL: leaf colour lightness, LA: leaf colour green/red, LB: leaf colour blue/yellow, FL: fruit colour lightness, FA: fruit colour green/red, FB: fruit colour blue/yellow, FLe: fruit length (mm), FWi: fruit width (mm), FW: fruit weight (g), PL: pedicel length (mm), PFB: proximal fruit blockiness, DFB: distal fruit blockiness, FST: fruit shape triangle, NL: number of locules, PMV: pollen morphological viability (%), PBV: pollen biological viability (%), SW: seed weight (g).
