# Supplementary material for: First interspecific multi-parent advanced generation inter-cross (MAGIC) population in Capsicum peppers: development, phenotypic evaluation, genomic analysis, and prospects
Source: Hortic Res. 2025 Jul 16;12(10):uhaf182. doi: 10.1093/hr/uhaf182 (PMC12537016; doi:10.1093/hr/uhaf182)
Supplement: Web_Material_uhaf182 [file web_material_uhaf182.zip › Supplementary Table 3.docx]

Supplementary table 3. Significant QTLs, chromosome, QTL start, QTL end, FDR adjusted value, statistical model, gene function, and metabolic pathway for stem colour, nodal anthocyanin, stem pubescence, filament colour, anthocyanin spot in the immature fruit, fruit colour at the immature stage, fruit colour at the mature stage, pedicel persistence, and fruit wall consistency.

| Chr | QTL start | QTL end | FDR-value | Statistical model | Gene function | Metabolic pathway |  |
| --- | --- | --- | --- | --- | --- | --- | --- |
| ***Stem colour*** | | | | | | | |
| chr10 | 200409946 | 200809947 | 4.44E-04 | BLINK | CPRD49 GDSL esterase/lipase | Lipid metabolism |  |
| chr10 | 200409946 | 200809947 | 4.44E-04 | BLINK | DEX1 Protein Defective in exine formation 1 | Pollen development |  |
| chr10 | 200409946 | 200809947 | 4.44E-04 | BLINK | OPS Protein Octopus | Root and vascular development |  |
|  | | | | | | | |
| ***Nodal anthocyanin*** | | | | | | | |
| chr10 | 91708986 | 92108987 | 5.29E-04 | BLINK | HSP17.5-M 17.5 kDa class I heat shock protein | Response to thermic shock |  |
| chr10 | 91708986 | 92108987 | 5.29E-04 | BLINK | PME46 pectinesterase/pectinesterase inhibitor 46 | Root development |  |
| chr10 | 185672100 | 186072101 | 4.68E-03 | MLMM | RPA1C Replication protein A 70 kDa DNA-binding subunit C | DNA replication |  |
| chr10 | 185672100 | 186072101 | 4.68E-03 | MLMM | SBT6.1 Subtilisin-like protease SBT6.1 | Cell development |  |
| chr10 | 185672100 | 186072101 | 4.68E-03 | MLMM | HEMH Ferrochelatase-2, chloroplastic | Hydrogen production |  |
| chr10 | 185672100 | 186072101 | 4.68E-03 | MLMM | CYCB2-4 Cyclin-B2-4 | Cell development |  |
| chr10 | 185672100 | 186072101 | 4.68E-03 | MLMM | PBL7 serine/threonine-protein kinase | Protein modification |  |
| chr10 | 185672100 | 186072101 | 4.68E-03 | MLMM | RPL4A 60S ribosomal protein L4-1 | Protein synthesis |  |
| chr10 | 185672100 | 186072101 | 4.68E-03 | MLMM | APRR7 Two-component response regulator-like | Response to temperature/circadian rhythm |  |
| chr10 | 185672100 | 186072101 | 4.68E-03 | MLMM | BTB/POZ domain-containing protein | Response to temperature/circadian rhythm |  |
| chr10 | 185672100 | 186072101 | 4.68E-03 | MLMM | TERC Thylakoid membrane protein, chloroplastic | Cell energy system |  |
|  | | | | | | | |
| ***Stem pubescence*** | | | | | | | |
| chr02 | 144824842 | 145224843 | 4.27E-02 | BLINK | CYS4 Cysteine proteinase inhibitor 4 | Response to general stress and general defence |  |
| chr02 | 144824842 | 145224843 | 4.27E-02 | BLINK | KIC Calcium-binding protein | Trichome morphogenesis |  |
| chr02 | 144824842 | 145224843 | 4.27E-02 | BLINK | Suberization-associated anionic peroxidase | General defence |  |
| chr02 | 144824842 | 145224843 | 4.27E-02 | BLINK | FAD-OXR Berberine bridge enzyme-like 22 | Oxidative reactions |  |
| chr02 | 144824842 | 145224843 | 4.27E-02 | BLINK | Berberine bridge enzyme-like 23 | Oxidative reactions |  |
| chr02 | 144824842 | 145224843 | 4.27E-02 | BLINK | G-type lectin S-receptor-like serine/threonine-protein kinase | Protein modification |  |
| chr02 | 144824842 | 145224843 | 4.27E-02 | BLINK | Pentatricopeptide repeat-containing protein | RNA modification process |  |
| chr10 | 232972943 | 233321800 | 4.19E-07 | MLMM | NSE4A | Chromosome integrity |  |
| chr10 | 232972943 | 233321800 | 4.19E-07 | MLMM | MOD1 Enoyl reductase, chloroplastic | Lipid metabolism |  |
| chr10 | 232972943 | 233321800 | 4.19E-07 | MLMM | Zinc finger CCCH domain-containing protein 14 | Response to general stress |  |
| chr10 | 232972943 | 233321800 | 4.19E-07 | MLMM | GMPM1 18 kDa seed maturation protein | Response to abiotic stress |  |
| chr10 | 232972943 | 233321800 | 4.19E-07 | MLMM | LEA46 Late embryogenesis abundant protein 46 | Response to abiotic stress |  |
| chr10 | 232972943 | 233321800 | 4.19E-07 | MLMM | R1B-17 Late blight resistance protein homolog | Pathogen defence |  |
| chr10 | 232972943 | 233321800 | 4.19E-07 | MLMM | R1A-3 Late blight resistance protein homolog | Pathogen defence |  |
| chr10 | 232972943 | 233321800 | 4.19E-07 | MLMM | Germin-like protein 2-2 | Plant and seed development |  |
| chr10 | 232972943 | 233321800 | 4.19E-07 | MLMM | Disease resistance protein | General defence |  |
| chr10 | 232972943 | 233321800 | 4.19E-07 | MLMM | R1B-23 Late blight resistance protein homolog | Pathogen defence |  |
| chr10 | 232972943 | 233321800 | 4.19E-07 | MLMM | Germin-like protein 2-3 | Plant and seed development |  |
| chr10 | 232972943 | 233321800 | 4.19E-07 | MLMM | R1A-10 Late blight resistance protein homolog | Pathogen defence |  |
| chr10 | 232972943 | 233321800 | 4.19E-07 | MLMM | Disease resistance protein | General defence |  |
| chr10 | 232972943 | 233321800 | 4.19E-07 | MLMM | PUB4 U-box domain-containing protein 4 | Protein degradation and ROS scavenging |  |
| chr10 | 232972943 | 233321800 | 4.19E-07 | MLMM | Disease resistance protein | General defence |  |
| chr10 | 232972943 | 233321800 | 4.19E-07 | MLMM | Disease resistance protein | General defence |  |
| chr10 | 232972943 | 233321800 | 4.19E-07 | MLMM | Phosphatase 2C 43 | Glucose metabolism |  |
| chr10 | 232972943 | 233321800 | 4.19E-07 | MLMM | RLP24 Ribosome biogenesis protein | Ribosome biogenesis |  |
| chr10 | 232972943 | 233321800 | 4.19E-07 | MLMM | LE25 Protein | Ion scavenger |  |
|  | | | | | | | |
| ***Filament colour*** | | | | | | | |
| chr10 | 194182140 | 194582141 | 3.60E-04 | MLMM | ELC Protein | Cell development |  |
| chr10 | 194182140 | 194582141 | 3.60E-04 | MLMM | D7OMT Isoflavone 7-O-methyltransferase | Formononetin biosynthesis |  |
| chr10 | 194182140 | 194582141 | 3.60E-04 | MLMM | FBL11 BTB/POZ domain-containing protein | Phenol accumulation |  |
| chr10 | 194182140 | 194582141 | 3.60E-04 | MLMM | CTR1 Serine/threonine-protein kinase | Ethylene signalling |  |
| chr10 | 194182140 | 194582141 | 3.60E-04 | MLMM | HEMA1 Glutamyl-tRNA reductase 1, chloroplastic | Photosynthetic metabolism |  |
| chr10 | 194182140 | 194582141 | 3.60E-04 | MLMM | IQM3 IQ domain-containing protein | Root development |  |
| chr10 | 194182140 | 194582141 | 3.60E-04 | MLMM | FBL11 BTB/POZ domain-containing protein | Phenol accumulation |  |
|  | | | | | | | |
| ***Anthocyanin spots*** | | | | | | | |
| chr04 | 219650264 | 220050265 | 6.22E-05 | BLINK | R1A Late blight resistance protein | Pathogen defence |  |
| chr06 | 5264024 | 5664025 | 2.20E-02 | BLINK | R1B-14 Late blight resistance protein homolog | Pathogen defence |  |
| chr06 | 5264024 | 5664025 | 2.20E-02 | BLINK | R1B-13 Late blight resistance protein homolog | Pathogen defence |  |
| chr06 | 5264024 | 5664025 | 2.20E-02 | BLINK | R1A-4 Late blight resistance protein homolog | Pathogen defence |  |
| chr06 | 5264024 | 5664025 | 2.20E-02 | BLINK | R1B-8 Late blight resistance protein homolog | Pathogen defence |  |
| chr06 | 5264024 | 5664025 | 2.20E-02 | BLINK | R1A-3 Late blight resistance protein homolog | Pathogen defence |  |
| chr06 | 5264024 | 5664025 | 2.20E-02 | BLINK | R1A-10 Late blight resistance protein homolog | Pathogen defence |  |
| chr06 | 5264024 | 5664025 | 2.20E-02 | BLINK | COX2 Cytochrome c oxidase subunit 2 | Cell energy system |  |
|  | | | | | | | |
| ***Fruit colour at intermediate stage*** | | | | | | | |
| chr10 | 7699760 | 8099761 | 1.17E-04 | BLINK/MLMM | LSH10 Protein light-dependent short hypocotyls 10 | Glucosinolates biosynthesis |  |
| chr10 | 7699760 | 8099761 | 1.17E-04 | BLINK/MLMM | PAB1 Proteasome subunit alpha type-2-A | Protein degradation |  |
| chr10 | 7699760 | 8099761 | 1.17E-04 | BLINK/MLMM | MENG 2-phytyl-1,4-beta-naphthoquinone methyltransferase, chloroplastic | DNA modification |  |
| chr10 | 7699760 | 8099761 | 1.17E-04 | BLINK/MLMM | TBL39 Protein trichome birefringence-like 39 | Cell development |  |
| chr10 | 7699760 | 8099761 | 1.17E-04 | BLINK/MLMM | OST3B dolichyl-diphosphooligosaccharide glycosyltransferase | Protein modification |  |
| chr10 | 7699760 | 8099761 | 1.17E-04 | BLINK/MLMM | PSL4 Glucosidase 2 subunit beta | Response to microorganisms |  |
| chr10 | 7699760 | 8099761 | 1.17E-04 | BLINK/MLMM | SD25 G-type lectin S-receptor-like serine/threonine kinase | Protein modification |  |
| chr10 | 7699760 | 8099761 | 1.17E-04 | BLINK/MLMM | 14-3-3-like protein B | Signalling |  |
| chr10 | 7699760 | 8099761 | 1.17E-04 | BLINK/MLMM | GTE4 Transcription factor G | Transcription factor |  |
| chr10 | 7699760 | 8099761 | 1.17E-04 | BLINK/MLMM | ACX3 Acyl-coenzyme A oxidase 3, peroxisomal | Lipid metabolism |  |
| chr10 | 20556647 | 20956648 | 6.96E-03 | BLINK | Endo-1,3;1,4-beta-D-glucanase | Abiotic stress response |  |
|  | | | | | | | |
| ***Fruit colour at mature stage*** | | | | | | | |
| chr01 | 4945937 | 5345938 | 1.38E-07 | BLINK | GT16 Xyloglucan-specific galacturonosyltransferase 1 | Root development |  |
| chr01 | 4945937 | 5345938 | 1.38E-07 | BLINK/MLMM | GSTT1 Glutathione S-transferase T1 | Protein degradation |  |
| chr01 | 4945937 | 5345938 | 1.38E-07 | BLINK/MLMM | HAL3A Phosphopantothenoylcysteine decarboxylase | Lipid metabolism |  |
| chr01 | 4945937 | 5345938 | 1.38E-07 | BLINK/MLMM | RALFL5 Protein RALF-like 5 | Hormone response and signalling |  |
| chr01 | 4945937 | 5345938 | 1.38E-07 | BLINK/MLMM | Ethylene-responsive proteinase inhibitor 1 | Fruit development and ripening |  |
| chr01 | 4945937 | 5345938 | 1.38E-07 | BLINK/MLMM | OSML81 Osmotin-like protein | Abiotic stress response |  |
| chr01 | 4945937 | 5345938 | 1.38E-07 | BLINK/MLMM | OSML13 Osmotin-like protein | Abiotic stress response |  |
| chr01 | 4945937 | 5345938 | 2.48E-03 | BLINK/MLMM | OSML15 Osmotin-like protein | Abiotic stress response |  |
| chr01 | 4945937 | 5345938 | 2.48E-03 | BLINK/MLMM | ERG3 Elicitor-responsive protein 3 | Abiotic stress response |  |
| chr01 | 4945937 | 5345938 | 2.48E-03 | BLINK/MLMM | MBD6 Methyl-CpG-binding domain-containing protein 6 | Transcription factor |  |
| chr04 | 229216238 | 229616239 | 2.38E-03 | BLINK/MLMM | CYP72A219 Cytochrome P450 | Cell energy system |  |
| chr04 | 229216238 | 229616239 | 2.38E-03 | BLINK/MLMM | F-box protein | Transcription factor |  |
| chr04 | 229216238 | 229616239 | 2.38E-03 | BLINK/MLMM | PCMP-E91 Pentatricopeptide repeat-containing protein | RNA modification process |  |
| chr04 | 229216238 | 229616239 | 2.38E-03 | BLINK/MLMM | TPS7, alpha-trehalose-phosphate synthase 7 | Glucose metabolism |  |
| chr04 | 229216238 | 229616239 | 2.38E-03 | BLINK/MLMM | SCPL19 Serine carboxypeptidase-like 19 | Protein degradation |  |
| chr04 | 229216238 | 229616239 | 2.38E-03 | BLINK/MLMM | SCPL52 serine carboxypeptidase-like 52 | Protein degradation |  |
| chr04 | 229216238 | 229616239 | 1.49E-02 | MLMM | SCPL17 Serine carboxypeptidase-like 17 | Protein degradation |  |
| chr06 | 232345051 | 232745052 | 2.09E-08 | BLINK | MOCS2 Molybdopterin synthase catalytic subunit | Plant growth |  |
| chr06 | 232345051 | 232745052 | 2.09E-08 | BLINK | CDKC-1 Cyclin-dependent kinase C-1 | Plant growth |  |
| chr06 | 232345051 | 232745052 | 2.09E-08 | BLINK | GAMYB Transcription factor | Flower and fruit development |  |
| chr06 | 232345051 | 232745052 | 2.09E-08 | BLINK | F-box/WD-40 repeat-containing protein | Transcription factor |  |
| chr06 | 232345051 | 232745052 | 2.09E-08 | BLINK | LTPG2 Non-specific lipid transfer protein GPI-anchored 2 | Lipid metabolism |  |
| chr06 | 232345051 | 232745052 | 2.09E-08 | BLINK | SE Serrate RNA effector molecule | RNA modification process |  |
| chr06 | 232345051 | 232745052 | 2.09E-08 | BLINK | SRM1 Transcription factor SRM1 | Transcription factor |  |
| chr06 | 232345051 | 232745052 | 2.09E-08 | BLINK | LSM8 Sm-like protein LSM8 | RNA modification process |  |
| chr06 | 232345051 | 232745052 | 2.09E-08 | BLINK | Glucan endo-1,3-beta-glucosidase 14 | Seed development |  |
| chr06 | 232345051 | 232745052 | 2.09E-08 | BLINK | EIN3 Protein ethylene insensitive 3 | Signalling |  |
| chr06 | 232345051 | 232745052 | 2.09E-08 | BLINK | Xyl2 Beta-xylosidase/alpha-L-arabinofuranosidase 2 | Protein degradation |  |
| chr06 | 232345051 | 232745052 | 2.09E-08 | BLINK | SMC3 Structural maintenance of chromosomes protein 3 | Chromosome structure |  |
| chr06 | 232345051 | 232745052 | 2.09E-08 | BLINK | FRS3 Protein far-1 related sequence 3 | Plant growth |  |
| chr06 | 232345051 | 232745052 | 2.09E-08 | BLINK | S13-6 G2/mitotic-specific cyclin | Cell development |  |
| chr06 | 232929162 | 233329163 | 1.81E-10 | BLINK | BLH1 BEL1-like homeodomain protein 1 | Embryo development |  |
| chr06 | 232929162 | 233329163 | 1.81E-10 | BLINK | PUB24 E3 ubiquitin-protein ligase | Response to microorganisms |  |
| chr06 | 232929162 | 233329163 | 1.81E-10 | BLINK | NTL8 NAC domain-containing protein 40 | Response to general stress |  |
| chr06 | 232929162 | 233329163 | 1.81E-10 | BLINK | CYP89A9 Cytochrome P450 89A9 | Cell energy system |  |
| chr06 | 232929162 | 233329163 | 1.81E-10 | BLINK | PSAO Photosystem I subunit O | Cell energy system |  |
| chr06 | 232929162 | 233329163 | 1.81E-10 | BLINK | mRNA-decapping enzyme-like protein | Epidermal morphology |  |
| chr06 | 232929162 | 233329163 | 1.81E-10 | BLINK | CCS Capsanthin/capsorubin synthase, chromoplastic | Carotenoid biosynthesis |  |
| chr06 | 232929162 | 233329163 | 1.81E-10 | BLINK | VAMP714 Vesicle-associated membrane protein 714 | Hormone response and signalling |  |
| chr06 | 232929162 | 233329163 | 1.81E-10 | BLINK | FAF3 Protein fantastic four 3 | Plant growth |  |
| chr06 | 232929162 | 233329163 | 1.81E-10 | BLINK | BAD1 Ankyrin repeat-containing protein | Plant growth |  |
| chr06 | 232929162 | 233329163 | 1.81E-10 | BLINK | FDX3 Ferredoxin-3, chloroplastic | Cell energy system |  |
| chr06 | 232929162 | 233329163 | 1.81E-10 | BLINK | Cytochrome b-c1 complex subunit 9 | Cell energy system |  |
| chr06 | 232929162 | 233329163 | 1.81E-10 | BLINK | FRI Protein frigida | Flower development and reproduction |  |
| chr06 | 232929162 | 233329163 | 1.81E-10 | BLINK | TGA9 Transcription factor | Flower development and reproduction |  |
| chr06 | 232929162 | 233329163 | 1.81E-10 | BLINK | LRX4 Leucine-rich repeat extensin-like protein 4 | Cell wall development |  |
|  | | | | | | | |
| ***Pedicel persistence*** | | | | | | | |
| chr10 | 228910007 | 229310008 | 3.86E-02 | MLMM | CYP96A15 Alkane hydroxylase MAH1 | Cell energy system |  |
| chr10 | 228910007 | 229310008 | 3.86E-02 | MLMM | Miraculin | General defence |  |
| chr10 | 228910007 | 229310008 | 3.86E-02 | MLMM | PIP5K4 Phosphatidylinositol 4-phosphate 5-kinase | Pollen development |  |
| chr10 | 228910007 | 229310008 | 3.86E-02 | MLMM | Thioredoxin-like 1-1, chloroplastic | Cell energy system |  |
| chr10 | 228910007 | 229310008 | 3.86E-02 | MLMM | TGA-2.1 TGACG-sequence-specific DNA-binding protein | Carotenoid biosynthesis |  |
| chr10 | 228910007 | 229310008 | 3.86E-02 | MLMM | SMG7 Protein | Meiosis |  |
| chr10 | 228910007 | 229310008 | 3.86E-02 | MLMM | HD16 Casein kinase 1-like protein | Flower development and reproduction |  |
| chr10 | 229862715 | 230262716 | 1.83E-05 | BLINK | PYM Protein | Cell division |  |
| chr10 | 229862715 | 230262716 | 1.83E-05 | BLINK | SAP8 Zinc finger A20; AN1 domain-containing stress-associated protein 8 | Response to general stress |  |
| chr10 | 229862715 | 230262716 | 1.83E-05 | BLINK | NEK6 Serine/threonine-protein kinase | Epidermal cell differentiation |  |
| chr10 | 229862715 | 230262716 | 1.83E-05 | BLINK | Vestitone reductase | Antioxidant isoflavone |  |
| chr10 | 229862715 | 230262716 | 1.83E-05 | BLINK | CRP1 Pentatricopeptide repeat-containing, chloroplastic | Cell and plant development |  |
| chr10 | 229862715 | 230262716 | 1.83E-05 | BLINK | DREB2F Dehydration-responsive element-binding protein | Transcription factor |  |
| chr10 | 229862715 | 230262716 | 1.83E-05 | BLINK | Zinc finger CCCH domain-containing protein 30 | RNA modification process |  |
| chr10 | 229862715 | 230262716 | 1.83E-05 | BLINK | REM4.2 Remorin 4.2 | Response to abiotic stress |  |
| chr10 | 229862715 | 230262716 | 1.83E-05 | BLINK | SAP11 Zinc finger AN1; C2H2 domain-containing stress-associated protein 11 | Response to general stress |  |
| chr10 | 229862715 | 230262716 | 1.83E-05 | BLINK | SMT1 Cycloartenol-C-24-methyltransferase | Lipid metabolism |  |
| chr10 | 229862715 | 230262716 | 1.83E-05 | BLINK | RRP45A Exosome complex component | RNA modification process |  |
| chr10 | 229862715 | 230262716 | 1.83E-05 | BLINK | TGA10 bZIP transcription factor | Response to abiotic stress |  |
| chr10 | 229862715 | 230262716 | 1.83E-05 | BLINK | DAO 2-oxoglutarate-dependent dioxygenase | Lipid metabolism |  |
| chr10 | 229862715 | 230262716 | 1.83E-05 | BLINK | NRPA1 DNA-directed RNA polymerase I subunit 1 | DNA replication |  |
| chr10 | 229862715 | 230262716 | 1.83E-05 | BLINK | ZIP4 Zinc transporter 4, chloroplastic | Zinc absorption |  |
| chr10 | 229862715 | 230262716 | 1.83E-05 | BLINK | PCMP-H51 Pentatricopeptide -containing protein, chloroplastic | RNA modification process |  |
| chr10 | 229862715 | 230262716 | 1.83E-05 | BLINK | Polygalacturonase | Fruit development and ripening |  |
|  | | | | | | | |
| ***Fruit wall consistency*** | | | | | | | |
| chr05 | 1634149 | 2034150 | 4.44E-02 | MLMM | G-type lectin S-receptor-like serine/threonine-protein kinase | Protein modification |  |
| chr05 | 1634149 | 2034150 | 4.44E-02 | MLMM | PGR3 Pentatricopeptide repeat-containing protein, chloroplastic/mitochondrial | RNA modification process |  |
| chr05 | 1634149 | 2034150 | 4.44E-02 | MLMM | MTACP2 Acyl carrier protein 2, mitochondrial | Lipid metabolism |  |
| chr05 | 1634149 | 2034150 | 4.44E-02 | MLMM | PGLP2 Phosphoglycolate phosphatase 2 | Glucose metabolism |  |
| chr05 | 1634149 | 2034150 | 4.44E-02 | MLMM | EMB2654 Pentatricopeptide repeat-containing protein | RNA modification process |  |
| chr05 | 1634149 | 2034150 | 4.44E-02 | MLMM | CHX28 Cation/H(+) antiporter 28 | Pollen development |  |
| chr05 | 1634149 | 2034150 | 4.44E-02 | MLMM | HSFB3 Heat stress transcription factor B-3 | Response to abiotic stress |  |
| chr05 | 1634149 | 2034150 | 4.44E-02 | MLMM | CCMB cytochrome c biogenesis, mitochondrial | Cell energy system |  |
| chr05 | 1634149 | 2034150 | 4.44E-02 | MLMM | RPL2 60S ribosomal protein L2, mitochondrial | DNA modification |  |
| chr05 | 1634149 | 2034150 | 4.44E-02 | MLMM | PIP5K1 Phosphatidylinositol 4-phosphate 5-kinase 1 | Lipid metabolism |  |
| chr05 | 1634149 | 2034150 | 4.44E-02 | MLMM | VLN2 Villin-2 | Cell development |  |
| chr05 | 1634149 | 2034150 | 4.44E-02 | MLMM | ABCA1 ABC transporter A family member 1 | Lipid metabolism |  |
| chr10 | 229862715 | 230262716 | 2.24E-02 | BLINK/MLMM | PYM Protein | Cell division |  |
| chr10 | 229862715 | 230262716 | 2.24E-02 | BLINK/MLMM | SAP8 Zinc finger A20; AN1 domain-containing stress-associated protein 8 | Response to general stress |  |
| chr10 | 229862715 | 230262716 | 2.24E-02 | BLINK/MLMM | NEK6 Serine/threonine-protein kinase | Epidermal cell differentiation |  |
| chr10 | 229862715 | 230262716 | 2.24E-02 | BLINK/MLMM | Vestitone reductase | Antioxidant isoflavone |  |
| chr10 | 229862715 | 230262716 | 2.24E-02 | BLINK/MLMM | CRP1 Pentatricopeptide repeat-containing protein, chloroplastic | Cell development |  |
| chr10 | 229862715 | 230262716 | 2.24E-02 | BLINK/MLMM | DREB2F Dehydration-responsive element-binding protein 2F | Transcription factor |  |
| chr10 | 229862715 | 230262716 | 2.24E-02 | BLINK/MLMM | Zinc finger CCCH domain-containing protein 30 | RNA modification process |  |
| chr10 | 229862715 | 230262716 | 2.24E-02 | BLINK/MLMM | REM4.2 Remorin 4.2 | Response to abiotic stress |  |
| chr10 | 229862715 | 230262716 | 2.24E-02 | BLINK/MLMM | SAP11 Zinc finger AN1; C2H2 domain-containing stress-associated protein 11 | Response to general stress |  |
| chr10 | 229862715 | 230262716 | 2.24E-02 | BLINK/MLMM | SMT1 Cycloartenol-C-24-methyltransferase | Lipid metabolism |  |
| chr10 | 229862715 | 230262716 | 2.24E-02 | BLINK/MLMM | RRP45A Exosome complex component | RNA modification process |  |
| chr10 | 229862715 | 230262716 | 2.24E-02 | BLINK/MLMM | TGA10 bZIP transcription factor | Response to microorganisms |  |
| chr10 | 229862715 | 230262716 | 2.24E-02 | BLINK/MLMM | DAO 2-oxoglutarate-dependent dioxygenase | Lipid metabolism |  |
| chr10 | 229862715 | 230262716 | 2.24E-02 | BLINK/MLMM | NRPA1 DNA-directed RNA polymerase I subunit 1 | DNA replication |  |
| chr10 | 229862715 | 230262716 | 2.24E-02 | BLINK/MLMM | ZIP4 Zinc transporter 4, chloroplastic | Zinc absorption |  |
| chr10 | 229862715 | 230262716 | 2.24E-02 | BLINK/MLMM | PCMP-H51 Pentatricopeptide repeat-containing protein, chloropl/mitochon | RNA modification process |  |
| chr10 | 229862715 | 230262716 | 2.24E-02 | BLINK/MLMM | Polygalacturonase | Fruit development and ripening |  |
| chr12 | 249642713 | 250042714 | 3.10E-02 | BLINK | matK Maturase K | RNA modification process |  |
| chr12 | 249642713 | 250042714 | 3.10E-02 | BLINK | FAD12 Delta(12)-acyl-lipid-desaturase | Lipid metabolism |  |
| chr12 | 249642713 | 250042714 | 3.10E-02 | BLINK | Short-chain type dehydrogenase/reductase | Lipid metabolism |  |
| chr12 | 249642713 | 250042714 | 3.10E-02 | BLINK | PDR1 Pleiotropic drug resistance protein 1 | Transcription factor |  |
| chr12 | 249642713 | 250042714 | 3.10E-02 | BLINK | AOP1.2 2-oxoglutarate-dependent dioxygenase | Glucosinolates biosynthesis |  |
| chr12 | 249642713 | 250042714 | 3.10E-02 | BLINK | AOP3 2-oxoglutarate-dependent dioxygenase | Glucosinolates biosynthesis |  |
| chr12 | 249642713 | 250042714 | 3.10E-02 | BLINK | FAD2 Delta(12)-fatty-acid desaturase | Lipid metabolism |  |
